# From Bifunctionality to Multifunctionality: Nitrogen-Doped Carbon Nanostructures (CN_*x*_) for Electrocatalytic Applications in Fuel Cells and Beyond

**DOI:** 10.1021/acs.chemmater.6c00472

**Published:** 2026-07-01

**Authors:** Anant Sohale, Snehal Patil, Seval Gunduz, Anne C. Co, Umit S. Ozkan

**Affiliations:** † William G. Lowrie Department of Chemical and Biomolecular Engineering, 2647 The Ohio State University, Columbus, Ohio 43210, United States; ‡ Department of Chemistry and Biochemistry, The Ohio State University, Columbus, Ohio 43210, United States

## Abstract

This article provides an overview of the studies performed in our laboratories for developing nitrogen-doped carbon nanostructures (CN_*x*_) as precious-metal-free bifunctional oxygen reduction reaction (ORR) and oxygen evolution reaction (OER) electrocatalysts for unitized regenerative fuel cells (URFCs). Additionally, our recent studies exploring the applications of these catalysts in halogen production via oxygen-depolarized cathode (ODC) technology have also been discussed. Future perspectives, challenges, and potential research avenues for exploring these materials as ‘multifunctional’ electrocatalysts have also been highlighted.

## Graphical abstract

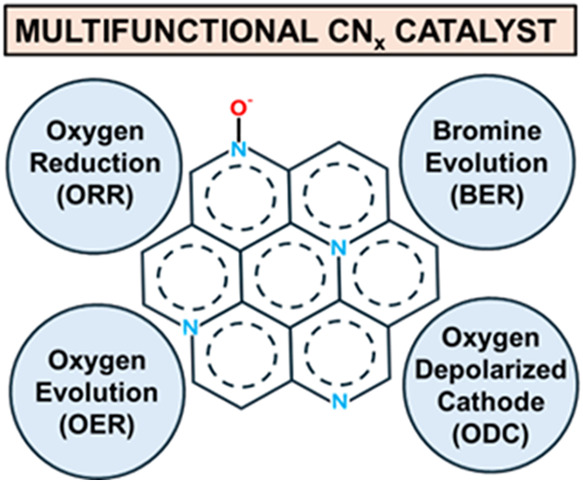

## 1 Background: Nitrogen-Doped Carbon Nanostructures (CN_*x*_) as ORR Electrocatalysts

There has been substantial focus on developing precious-metal-free alternatives to state-of-the-art Pt-based electrocatalysts for the oxygen reduction reaction (ORR) in the last few decades.^1,2^ Nitrogen-doped carbon nanostructures (CN_*x*_) have emerged as a promising class of materials for fuel cell applications.^3,4^ The CN_*x*_-type materials have not only served as excellent support materials for efficiently dispersing and stabilizing metal nanoparticles in supported metal catalysts,^5,6^ but have also exhibited significant intrinsic ORR activity in the absence of an active metal.^7–19^ Ozkan and co-workers were the first group to demonstrate that precious-metal-free CN_*x*_ materials were active for ORR in acidic medium, making them promising catalysts for proton exchange membrane (PEM) fuel cells.^10,13^ A plethora of methods have been reported in the literature to synthesize CN_*x*_ catalysts.^20,21^ Chemical vapor deposition (CVD) is one of the most commonly adopted methods for the synthesis of CN_*x*_-type materials.^22^ Although the specifics of CVD-type processes vary, the synthesis schemes remain broadly similar. The initial step involves the preparation of growth substrates or templates on which carbon nanostructures are grown by pyrolysis of carbon- and nitrogen-containing precursors.^12,23^ The metal-based growth substrates are then removed by washing with mineral acids, yielding CN_*x*_ catalysts free of any surface metal. The Ozkan group prepared growth substrates using commercial carbon (Vulcan carbon) and oxide supports (Al_2_O_3_, SiO_2_, MgO, etc.) doped with metal (for catalyzing nanostructure formation).^13,24^ Acetonitrile was used as the carbon and nitrogen source in the pyrolysis step.^12^ Ozkan and co-workers also conducted in situ X-ray absorption spectroscopy (XAS) for understanding the nanostructure growth process and changes in oxidation state of the metal precursor during acetonitrile pyrolysis.^23^ In their initial studies, CN_*x*_-type materials were synthesized by using various combinations of oxide/carbon supports and metal dopants, and their ORR activities were compared. The nature of support and metal dopants (Co, Fe, Ni, etc.) controlled the nanogeometry and the structural properties of carbon materials formed.^9,11–13,25,26^ Figure 1 shows the carbon nanostructures with various nanogeometries.

**Figure 1:**
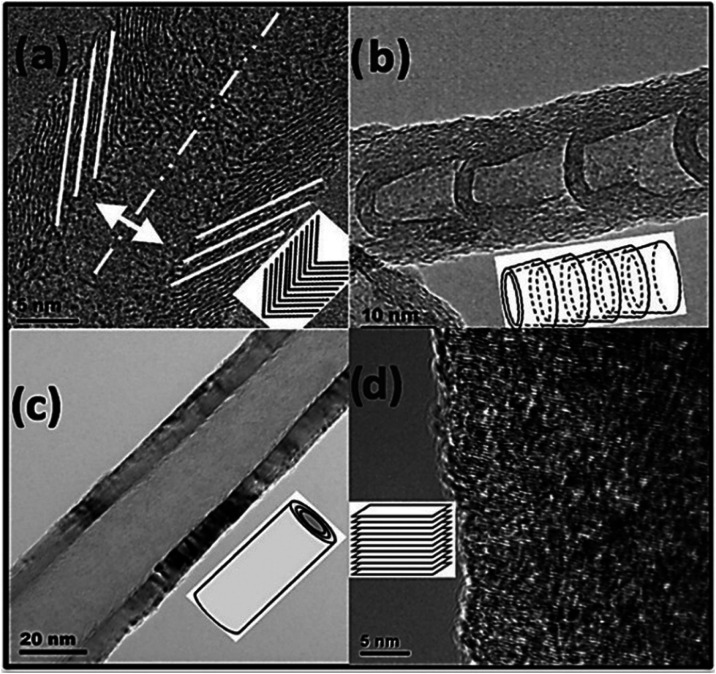
Various carbon nanogeometries and their TEM images: (a) herringbone, (b) stacked cup, (c) multiwalled nanotube, and (d) stacked platelet [adapted with permission from ref 26 Copyright 2014. Springer Nature].

The incorporation of nitrogen in these nanostructures led to the formation of various nitrogen (N) moieties such as quaternary-N (graphitic-N), pyridinic-N, and pyridinic-N oxide (Figure 2a), commonly identified using ex situ X-ray photoelectron spectroscopy (XPS).^27,28^ The ORR activity of these catalysts was found to be correlated with the relative pyridinic-N content (Figure 2b), which preferentially formed on the edge planes.^29^

**Figure 2:**
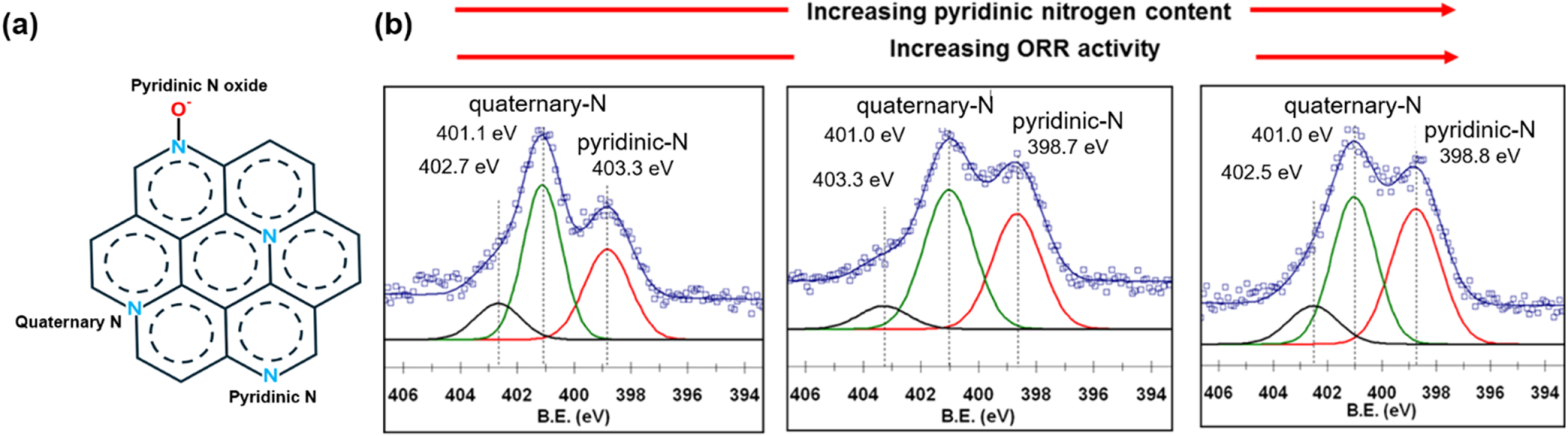
(a) Visual representation of various N sites in CN_*x*_. (b) N 1s XPS of CN_*x*_ catalysts with varying relative pyridinic-N content [adapted with permission from ref 26 Copyright 2014 Springer Nature].

Modulation of the pyridinic-N content by controlling the edge plane exposure or creation of active sites via an additional heteroatom dopant provided the rationale for enhancing the ORR performance of CN_*x*_ catalysts. Various strategies for synthesis modifications, such as regulation of pyrolysis temperature or doping additional heteroatoms (halogen, sulfur, phosphorus, boron, etc.), were shown to affect the pyridinic-N content, change the electronic distribution of these catalysts, or aid the growth of carbon nanostructures.^30–37^ However, it was still not clear what exactly constituted the active sites in the CN_*x*_ catalysts. Moreover, since the synthesis process to grow the carbon–nitrogen nanostructures in CN_*x*_ catalysts involved the presence of a transition metal for catalyzing the formation of nanostructures,^24^ the possibility of residual traces of transition metal after the acid washing step contributing toward the observed ORR activity of CN_*x*_ materials became a topic of extensive debate.^38,39^ Furthermore, the development of metal-coordinated nitrogen–carbon or MeNC-type materials (where Me = Fe, Co, etc.) as ORR catalysts with somewhat similar synthesis procedures and transition-metal-based active sites^40–47^ aggrandized the claims regarding the existence of metal-based active sites in CN_*x*_ catalysts.

To resolve this controversy, Dai and co-workers prepared nitrogen-doped carbon catalysts without using any metal-based precursors and showed significant ORR activity of N-doped carbon in both acidic and alkaline media.^48^ Similarly, Matter et al. also synthesized CN_*x*_ catalysts with considerable ORR activity, without using any transition metal dopants.^9^ However, it was still essential to settle the debate on the likelihood of contributions from possible traces of metal in CN_*x*_ catalysts prepared by using metal-based growth substrates. To address these concerns, Ozkan and co-workers conducted ORR poisoning studies with known metal poisons such as chlorides, cyanides, hydrogen sulfide, and carbon monoxide to further discern the absence of metal-based active sites in CN_*x*_ catalysts. Figure 3 highlights the poisoning studies conducted by the Ozkan group, using half-cell measurements and spectroscopy techniques. Catalysts with metal-based active sites showed strong ORR poisoning (diminished ORR activity), while CN_*x*_ catalysts exhibited remarkable tolerance to these metal poisons.^49–53^ Ex situ characterization studies corroborated the electrochemical measurements, showing strong interaction/formation of bonds with poison molecules in metal-based catalysts, while no interaction was observed for CN_*x*_ catalysts. Other research groups also observed similar ORR poisoning tolerance of metal-free N sites in their catalysts, while metal-based active sites were deactivated due to the poison molecules.^54–57^ Cherif et al. used fluorination to poison the FeNC/CN_*x*_-type catalysts and observed that Fe sites showed complete deactivation while metal-free N sites were found to be immune to poisoning by fluorination.^58^ These ORR poisoning studies confirmed that metal was indeed not a part of the ORR active sites in the CN_*x*_ materials.

**Figure 3:**
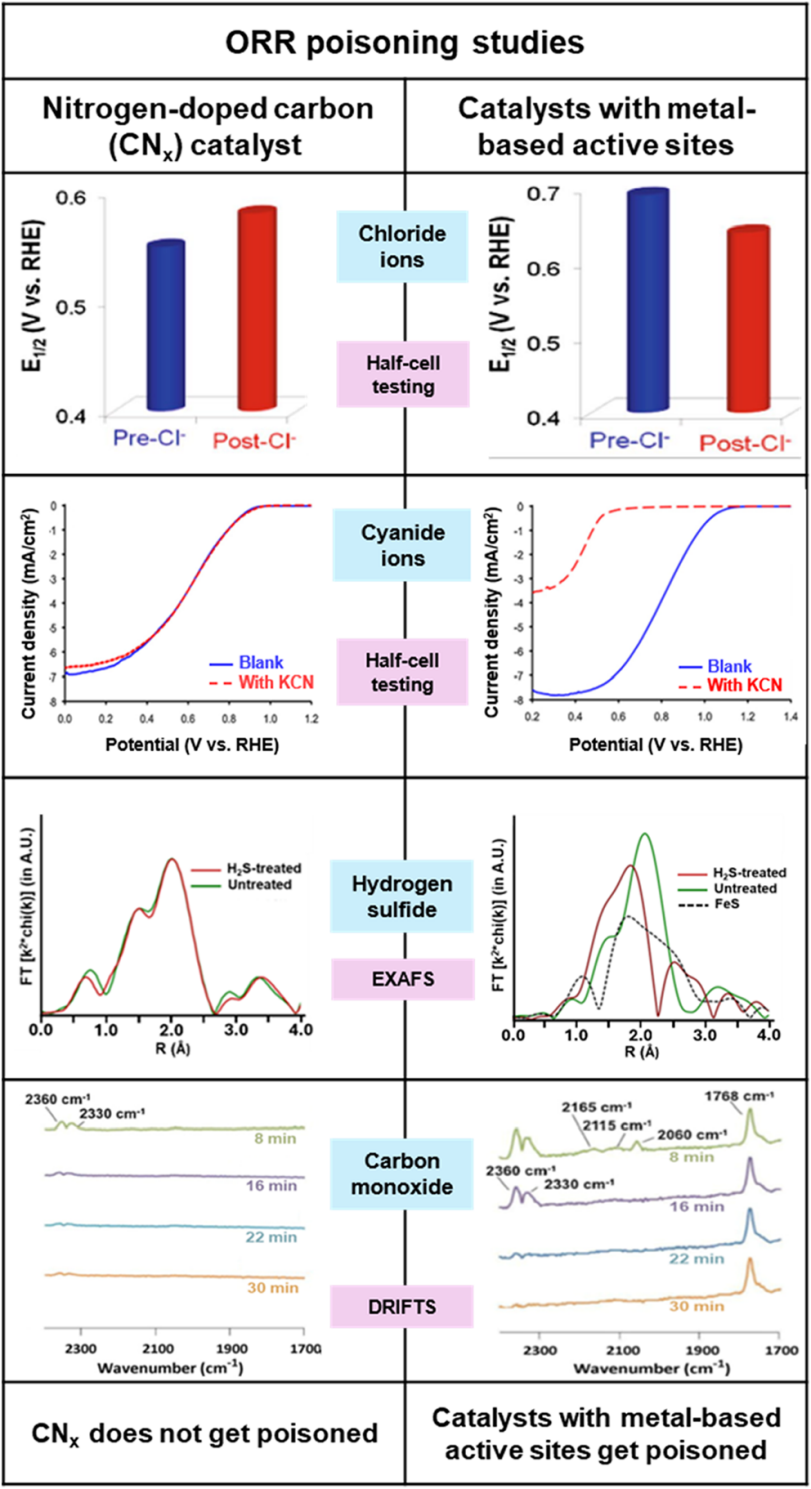
Schematic summarizing the poisoning studies to ascertain the absence of metal-based active sites in CN_*x*_ catalysts [Adapted with permission from ref 50 Copyright 2012 Elsevier. Adapted from ref 51 Copyright 2014 American Chemical Society. Adapted with permission from ref 53 Copyright 2017 Springer Nature. Adapted with permission from ref 26 Copyright 2014 Springer Nature].

Besides providing insights into the nature of active sites, the poisoning studies had wide-ranging implications for developing robust catalysts that are tolerant to deactivation effects. For example, poisoning resistance toward CO is desirable in fuel cells, which is commonly found as an impurity in H_2_ streams in anodic compartments. Crossover of even trace amounts of CO from the anode compartment to the cathode compartment can severely poison metal-based cathode catalysts.^59^ Moreover, CN_*x*_-type catalysts also exhibited remarkable ORR poisoning tolerance for methanol, which is essential for developing robust ORR catalysts resistant to deactivation by crossover of methanol from the anodic compartment in direct methanol fuel cell applications.^60^ Hence, the excellent poisoning resistance of CN_*x*_ demonstrated its potential as a poisoning-resistant, robust ORR catalyst and ruled out the presence of metal-based active sites.

To further prove that CN_*x*_ and FeNC did not have identical active sites, Ozkan group conducted systematic studies exploring the influence of acid washing on CN_*x*_ and FeNC catalysts. The acid washing step is crucial to remove any exposed metal on the CN_*x*_ surface. However, the synthesis of FeNC catalysts does not involve acid washing step, and thus the active Fe sites remain intact on the surface and accessible to O_2_ molecules. Acid washing of an unwashed material with ORR- active Fe species would leach the Fe species, leading to a decline in ORR activity.^61^ Figure 4 briefly summarizes the studies conducted by the Ozkan group to investigate the role of acid washing in distinguishing the nature of active sites in CN_*x*_ and FeNC materials. It was evident that the acid washing step led to an increase in ORR activity compared to unwashed samples for CN_*x*_, while the opposite effect was observed in FeNC catalysts.^62^ In CN_*x*_, increased activity was primarily attributed to the removal of inactive metal particles and/or nonconductive metal oxide support, while the loss in activity of FeNC was likely due to the loss of ORR active Fe species.^62,63^ Ex situ superconducting quantum interference device (SQUID) magnetometry also showed a drastic increase in saturation magnetization after acid washing due to removal of diamagnetic MgO phase in CN_*x*_, while a decrease was observed in FeNC catalysts, likely as a result of loss of zerovalent Fe.^62,63^ Additionally, ex situ Mössbauer and extended X-ray absorption fine structure (EXAFS) spectroscopy showed drastic changes in the type of Fe species in CN_*x*_ catalysts due to acid washing. The Fe species in CN_*x*_ catalysts changed from oxide form to predominantly carbide and metallic form, post acid washing.^61,62^ Additionally, ex situ transmission electron microscopy (TEM) images also depicted that residual traces of metal species were encased in the bulk structure of CN_*x*_, making them inaccessible for O_2_ molecules. In contrast, only the relative abundance of Fe species was affected by acid washing in FeNC catalysts, and not the nature of Fe species. Moreover, site density calculations using Mössbauer spectroscopy showed a reduction in planar FeN_4_ sites (hypothesized as ORR active sites in FeNC catalysts)^46^ post acid washing, indicating that they were possibly getting leached away due to acid washing, resulting in the observed loss of ORR activity.^61^ These results, in conjunction with poisoning studies, provided further evidence that ORR active sites did not have a metal center in CN_*x*_ and highlighted that FeNC and CN_*x*_ were indeed different classes of materials with different types of ORR active sites. However, the possibility of an electronic/charge transfer effect due to the encased residual metal indirectly contributing to the ORR active site in CN_*x*_ catalysts is not ruled out.^38,64^ Nevertheless, it should also be noted that multiple studies have demonstrated significant ORR activity of CN_*x*_ catalysts prepared without any metal, underscoring that the presence of metal is not necessary for ORR with CN_*x*_ catalysts.^9,48^

**Figure 4:**
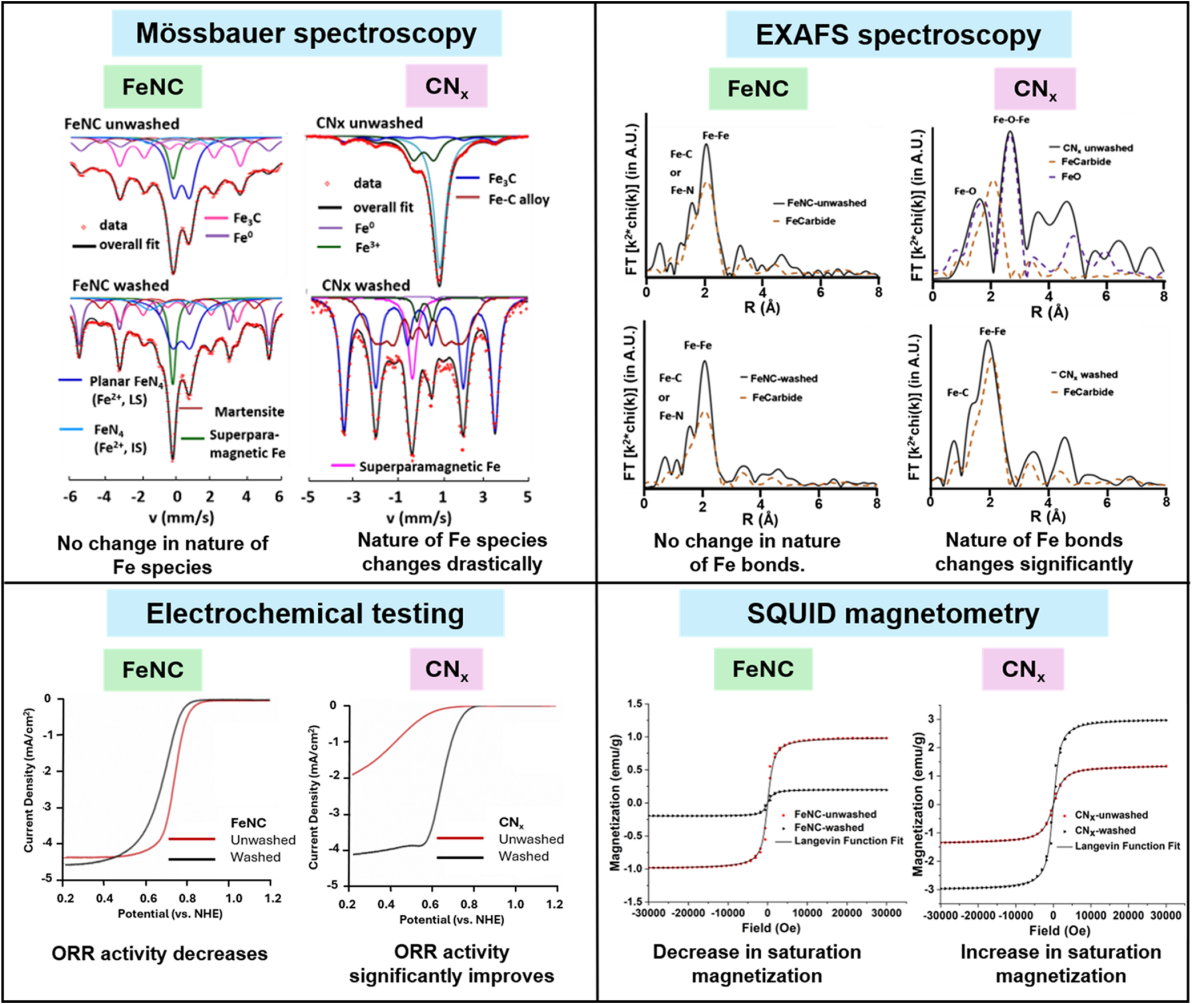
Distinguishing the nature of ORR active sites in CN_*x*_ and FeNC catalysts using acid washing [adapted with permission from ref 61 Copyright 2016 Springer Nature. Adapted with permission from ref 62 Copyright 2014 Elsevier. Adapted from ref 63 Copyright 2018 American Chemical Society].

Although the debate of CN_*x*_ vs FeNC was largely settled, there was still no clear consensus on what exactly constituted the ORR active site in CN_*x*_. Ozkan’s group and many other research groups hypothesized that pyridinic-N was either the active site or a marker of the active site.^28,65–68^ Guo et al. used precisely doped nitrogen in highly oriented pyrolytic graphite (HOPG) catalysts with different pyridinic-N content and found a correlation of ORR performance with pyridinic-N content.^65^ Ishikawa and co-workers also had similar observations in vertically aligned N-doped carbon nanotubes.^68^ Xing et al. used post reaction synchrotron XPS to show that carbon atoms adjacent to pyridinic-N were the primary active sites.^67^ In contrast, there were also studies claiming that graphitic-N could also be one of the possible active sites or its marker, especially in alkaline conditions.^69,70^ Chen and co-workers showed that graphitic-N was responsible for ORR activity in alkaline KOH solution using activity attenuation studies and density functional theory (DFT) calculations.^70^ Behan and co-workers studied the pH dependence of N species and found that pyridinic-N concentration correlated with the ORR activity in acidic media and with graphitic-N in alkaline conditions.^71^

The differing views of the precise nature of active sites made the rational design and tailoring of the CN_*x*_ catalysts highly challenging. Moreover, there was still no known molecule that could selectively poison the ORR active site in CN_*x*_ catalysts and provide crucial insights into the identity of ORR active sites in CN_*x*_. In 2016, Ozkan and co-workers were the first to report that phosphate anions could poison the ORR on CN_*x*_ catalysts. The study proposed two active site models: (i) pyridinic-N that became inactive by protonation or (ii) carbon adjacent to pyridinic-N rendered inactive by strongly adsorbed phosphate anion.^66^ Subsequently, Wang and co-workers also conducted ORR poisoning studies by selectively grafting acetyl groups on pyridinic-N/carbon adjacent to it (ortho carbon). They observed complete activity loss only in the latter case (ortho carbon) and concluded that the carbon neighboring pyridinic-N was the ORR active site. This was also supported by DFT calculations, which confirmed favorable O_2_ adsorption on the ortho carbon.^72^ Recently, another study from the Ozkan group showed that in situ bubbling of CO_2_ could also partially poison ORR on CN_*x*_ via the formation of carbonate and bicarbonate ions.^73^

In addition to pyridinic-N/graphitic-N content, other factors such as conductivity, porosity, and nature of defects could also affect ORR activity.^57,74^ Heteroatom doping influences the conductivity of the carbon framework, which may improve the charge transfer characteristics and enhance electrocatalytic activity.^75^ However, higher conductivity may not directly translate to superior ORR activity. Matter et al. observed that CN_*x*_ prepared from Fe/MgO supports had higher ORR activity compared to commercial Vulcan carbon, despite having slightly lower conductivity than Vulcan carbon.^9^ Although catalysts with higher edge plane exposure (stacked cup/herringbone nanogeometries) exhibited higher ORR activity, the nanostructure itself was not the factor directly contributing to higher ORR activity. Higher edge plane exposure (due to the nanogeometry) merely provided appropriate sites for nitrogen to get incorporated into the graphitic carbon matrix.^29^ Another important and comprehensively discussed factor that can influence the ORR activity of CN_*x*_ catalysts is the nature and degree of the defects. Some studies have even claimed that specific kinds of defects (such as pentagon defects) could be primarily responsible for ORR activity.^76^ Formation of defects and specific heteroatom (nitrogen) species (such as pyridinic-N) is often considered to be interdependent on each other, and their individual contributions toward the ORR activity of CN_*x*_ catalysts are difficult to separate. Zhu et al. demonstrated the synergistic effects of N-doping and pentagon defects.^77^ Precisely constructed graphitic-N coordinated with pentagon defects exhibited significant ORR activity due to favorable binding energy with ORR intermediates in both acidic and alkaline media.^77^ There has been recent focus on decoupling the influence of heteroatom doping and defects to clarify their individual contributions toward ORR activity. Jia et al. reported that pentagon-type defects induced by controlled pyridinic-N-doping followed by removal of N-dopants in model HOPG catalysts (referred to as D-HOPG) exhibited excellent ORR activity.^78^

Although these studies have provided considerable insights into the nature of active sites and the origin of ORR activity in CN_*x*_-type catalysts, a clear and comprehensive active site model supported by both theory and experiment still remains an ongoing research area.

In addition to its potential as an inexpensive ORR catalyst, applications of CN_*x*_ for other electrochemical reactions, including the OER, have also emerged. Bifunctional ORR/OER electrocatalysis on CN_*x*_-type catalysts has been discussed in numerous review articles,^79–82^ but the focus has largely been restricted to energy conversion/storage devices such as fuel cells, electrolyzers, and batteries. In this paper, we uniquely highlight the potential multifunctionality of CN_*x*_ materials with applications such as halogen production, extending beyond conventional ORR/OER. We also provide future perspectives on emerging applications of CN_*x*_-type catalysts in the field of electrosynthesis as well as the challenges in developing these materials as multifunctional electrocatalysts.

## 2 CN_*x*_ as Bifunctional Electrocatalysts for Applications in Unitized Regenerative Fuel Cells

### 2.1 Unitized Regenerative Fuel Cells (URFCs)

The growing demand for energy, limited availability of conventional energy sources, and stringent environmental regulations have driven research toward renewable and clean energy sources such as solar and wind energy.^83^ These renewable energy sources often suffer from intermittency in energy supply, hampering their large-scale operation.^84^ Coupling them with auxiliary energy conversion and storage devices, such as URFCs, can enhance grid resilience and overcome their seasonality and intermittency.^85^

URFC can operate as a fuel cell as well as an electrolyzer. The fuel cell mode involves the conversion of chemical energy (usually from hydrogen gas) into electrical energy. In PEMFCs, hydrogen oxidation reaction (HOR) takes place at the anode and oxygen reduction reaction (ORR) at the cathode as represented in eqs 1 and 2.^86^ Water being the only byproduct, the PEMFCs are claimed to have nearly zero carbon emissions.

1 $${\mathrm{cathode}:\;O}_{2}+4H^{+}+4e^{-}\to 2H_{2}O,\left(E^{o}=1.23\;V\;\mathrm{vs}\;\mathrm{SHE}\right)$$

2 $${anode:\;2H}_{2}\to 4H^{+}+4e^{-}\left(E^{o}=0\;V\;vs\;SHE\right)$$

In the electrolyzer mode, electrical energy is used to produce hydrogen gas from water. In a PEM electrolyzer, hydrogen evolution reaction (HER) occurs at the cathode and oxygen evolution reaction (OER) occurs at the anode (eqs 3 and 4).^87^

3 $${\mathrm{cathode}:\;4H^{+}+4e^{-}\to 2H}_{2}$$

4 $${\mathrm{anode}:\;2H_{2}O\to O}_{2}+4H^{+}+4e^{-}$$

The sluggish kinetics of ORR and OER necessitate widespread use of precious-metal-based cathode catalysts. Hence, the efficient operation of URFCs in both fuel cell and electrolyzer modes requires highly active “bifunctional” electrocatalysts that can catalyze both the bottleneck reactions (ORR and OER). However, the state-of-the-art electrocatalysts for ORR and OER (platinum and iridium, respectively),^88,89^ usually lack the critical bifunctionality.^90^ Moreover, traditional carbon-supported catalysts, though common in URFCs, undergo oxidation at higher oxidative potentials.^91^ Since pure Pt and Ir metal-based catalysts show poor bifunctional activity toward OER/ORR, Ir-incorporated bimetallic Pt catalysts have also been developed, but they still suffer from economic challenges.^92,93^ To overcome the economic challenges of precious-metal-based catalysts, consistent efforts have been undertaken to develop bifunctional precious-metal-free catalysts for URFC applications. M-NC catalysts (where M = Fe, Co, Mn), spinel metal oxides (such as Co_*x*_Mn_3‑x_O_4_, NiCo_2_O_4_), Ni-, Co-based sulfides on carbon-based supports, alloyed metals on heteroatom-doped carbon, perovskite oxides (such as CaMn_7_O_12_), hierarchical carbon materials, and some transition metal phosphides/oxides have been widely explored as potential bifunctional ORR/OER electrocatalysts.^47,94–99^ Metal-free CN_*x*_ catalysts also exhibited significant ORR and OER activity, making them suitable candidates for bifunctional electrocatalysis.^35,82^

### 2.2 ORR/OER Performance Metrics

The ORR/OER activity of CN_*x*_-type catalysts is compared using background-corrected polarization curves obtained in a rotating disk electrode (RDE) half-cell setup. The following performance metrics have been used for RDE tests (i) OER overpotential (η_*OER*_): defined as the additional potential (over *E*° = 1.23 V) that was applied to achieve an OER current density of 10 mA/cm_geometric_^2^ ^35,47^ (ii) ORR overpotential (η_ORR_): defined as the additional potential (over *E*° = 1.23 V) that was applied to achieve an ORR current density of −3 mA cm_geometric_^2^ ^47,98^ (iii) kinetic current density for ORR: an indicator of intrinsic electrochemical activity, free from mass transfer effects. For ORR, kinetic current density is obtained from the Koutecky–Levich equation (usually defined at a particular voltage in the region of 0.7–0.8 V vs RHE) (iv) half-wave potential (*E*_1/2_) for ORR: The potential at which the ORR current density becomes half of the limiting value (v) selectivity for ORR via 4-electron transfer to H_2_O: The number of electrons transferred in ORR obtained from rotating ring disk electrode (RRDE) technique or Koutecky–Levich analysis.^33^ (vi) The bifunctional electrocatalytic activity is measured by comparing the total overpotential requirements for OER and OER (η_total_). The total overpotential (η_total_) is given by eq 5.^35^

5 $$\;total\;overpotential\;\left(\eta_{\mathrm{total}}\right)=\eta_{OER}+\eta_{ORR}$$

The stability (durability) of catalysts is determined by comparing the relative changes in these performance metrics after potential holding/cycling for long durations/cycles. These performance metrics show strong correlation with structural/morphological descriptors of CN_*x*_-type catalysts, such as defects, distribution of nitrogen species, porosity, and even the nature of the catalyst film.^17,35,74^

Herein, we discuss the studies conducted in the Ozkan group on the bifunctionality of CN_*x*_ catalysts for URFC applications.

### 2.3 Comparison of Bifunctionality of CN_*x*_ with State-of-the-Art Catalysts

The bifunctional activity behavior of CN_*x*_ for ORR and OER was investigated by Mamtani et al.^35^ Activity measurements were conducted using O_2_-saturated acidic electrolytes in a conventional rotating disk electrode (RDE) half-cell system. Figure 5a depicts the ORR cathodic polarization curves of CN_*x*_ compared with Pt/C and Ir/C catalysts. The ORR onset potentials (potential at which background-corrected current density reached a value of −0.1 mA/cm_geometric_^2^) followed the order of Pt/C > CN_*x*_ > Ir/C. Both the half-wave potential and potential at −3 mA/cm_geometric_^2^ indicated that Ir/C (the state-of-the-art OER catalyst) was the least active for ORR, while Pt/C exhibited the highest ORR activity, followed by CN_*x*_. All of the catalysts showed selectivity (number of electrons transferred, n) of approximately 4, confirming predominant transformation of O_2_ to H_2_O.

**Figure 5:**
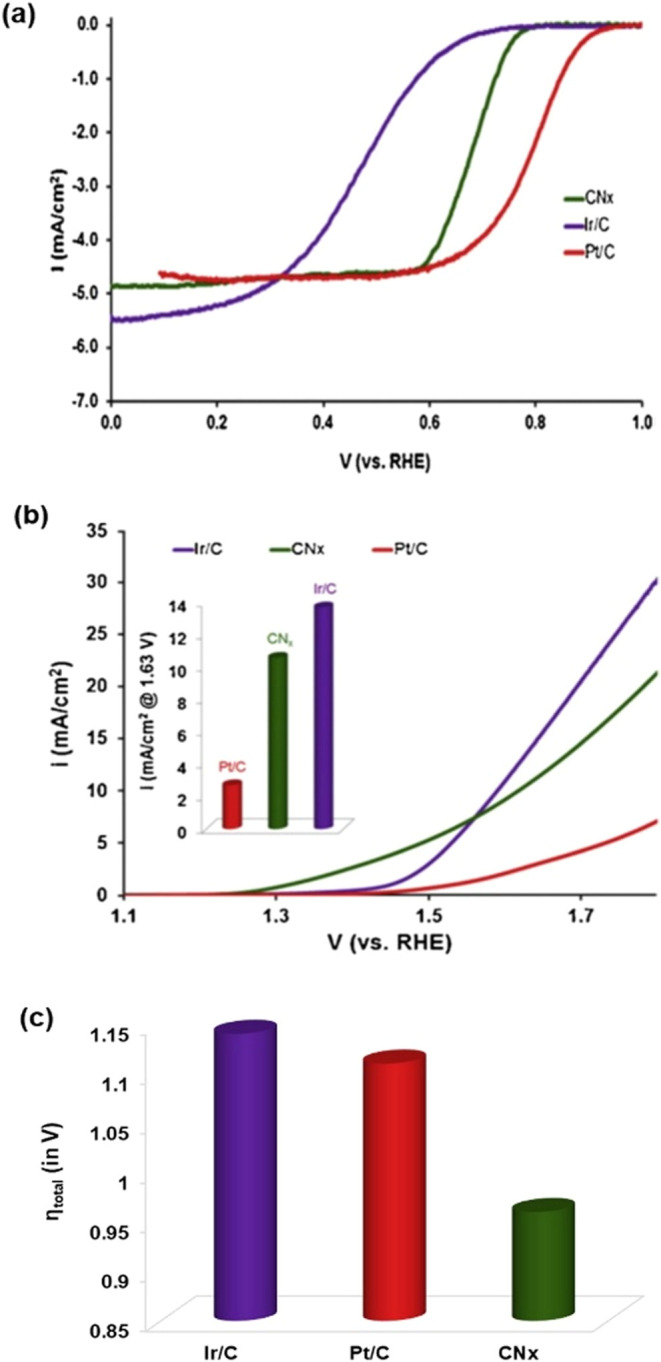
(a) ORR cathodic polarization curves, (b) OER anodic polarization curves (inset: OER current density at 1.63 V_RHE_), and (c) total overpotential for ORR and OER of CN_*x*_, commercial Pt/C, and commercial Ir/C in a rotating disk electrode (RDE) system [Adapted with permission from ref 35 Copyright 2018 Elsevier].

The OER activity of CN_*x*_ was measured by using anodic polarization curves and was also compared with commercial Pt/C and Ir/C samples, as shown in Figure 5b. CN_*x*_ demonstrated the lowest overpotential, followed by Ir/C and Pt/C. When the current densities were compared at 1.63 V, the activity order was Ir/C > CN_*x*_ ≫ Pt/C. A common metric used for comparison of OER activity, which is the potential at which OER current density becomes 10 mA/cm_geometric_^2^, also indicated that Pt/C (state-of-the-art ORR catalyst) is the least active (1.87 V) for OER, while CN_*x*_ showed similar potential compared to Ir/C (1.62 and 1.59 V, respectively).

Figure 5c depicts the comparison of η_total_ for CN_*x*_, Pt/C, and Ir/C. CN_*x*_ demonstrated the lowest total overpotential requirement of 0.96 V while Pt/C and Ir/C showed similar but higher overpotential requirements of 1.11 and 1.14 V, respectively. This highlights the excellent potential of CN_*x*_ as a bifunctional electrode in URFCs (Figure 6).

**Figure 6:**
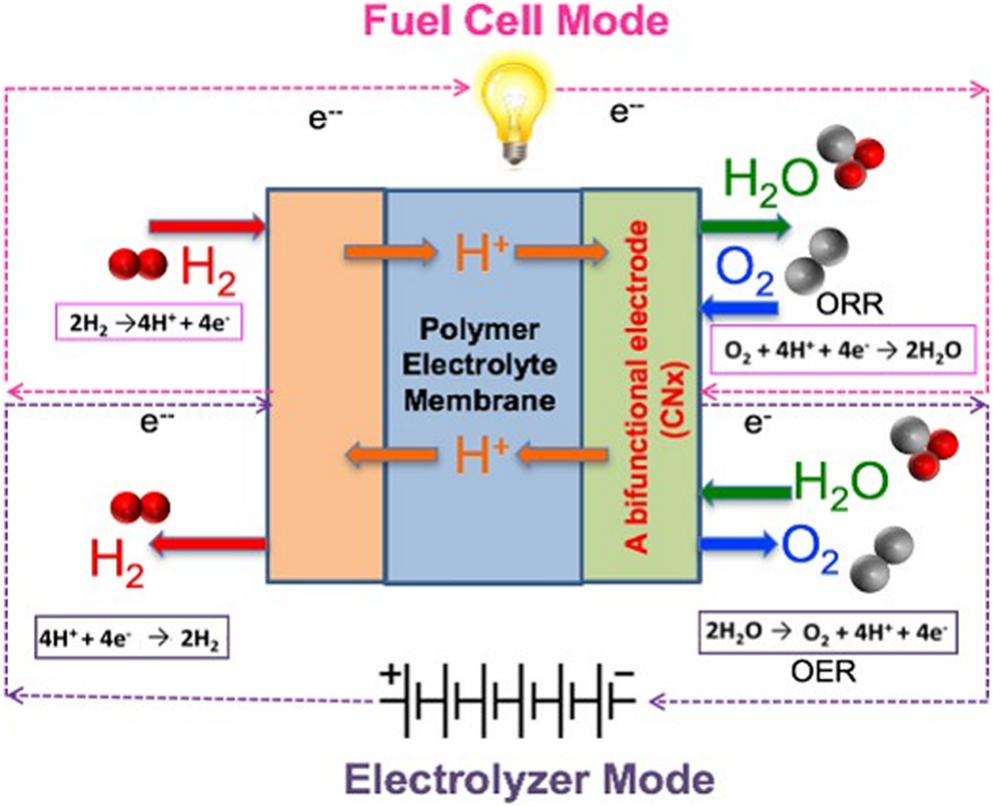
Unitized regenerative fuel cell (URFC) with CN_*x*_ as a bifunctional electrode for ORR and OER [Reproduced with permission from ref 35 Copyright 2018 Elsevier].

### 2.4 Effect of Pyrolysis Temperature and Type of Nitrogen Species on the Bifunctional Activity of CN_*x*_

After benchmarking the activity of CN_*x*_ with that of state-of-the-art catalysts, it was important to understand the influence of various N species on bifunctionality. Mamtani et al. synthesized CN_*x*_ catalysts with different distribution of nitrogen species by pyrolyzing the material at different temperatures: 750 °C, 800 °C, 850 °C, and 900 °C.^35^ The surface atomic composition of C, O, and N, analyzed using ex situ XPS, remained nearly the same for the CN_*x*_ catalysts pyrolyzed at different temperatures. However, the distribution of nitrogen species, namely, pyridinic-N, quaternary-N, and pyridinic-N^+^O^–^, was different on these catalysts. Increasing pyrolysis temperature resulted in a relative increase in the composition of pyridinic-N species while the relative composition of quaternary-N species decreased (Figure 7a).

**Figure 7:**
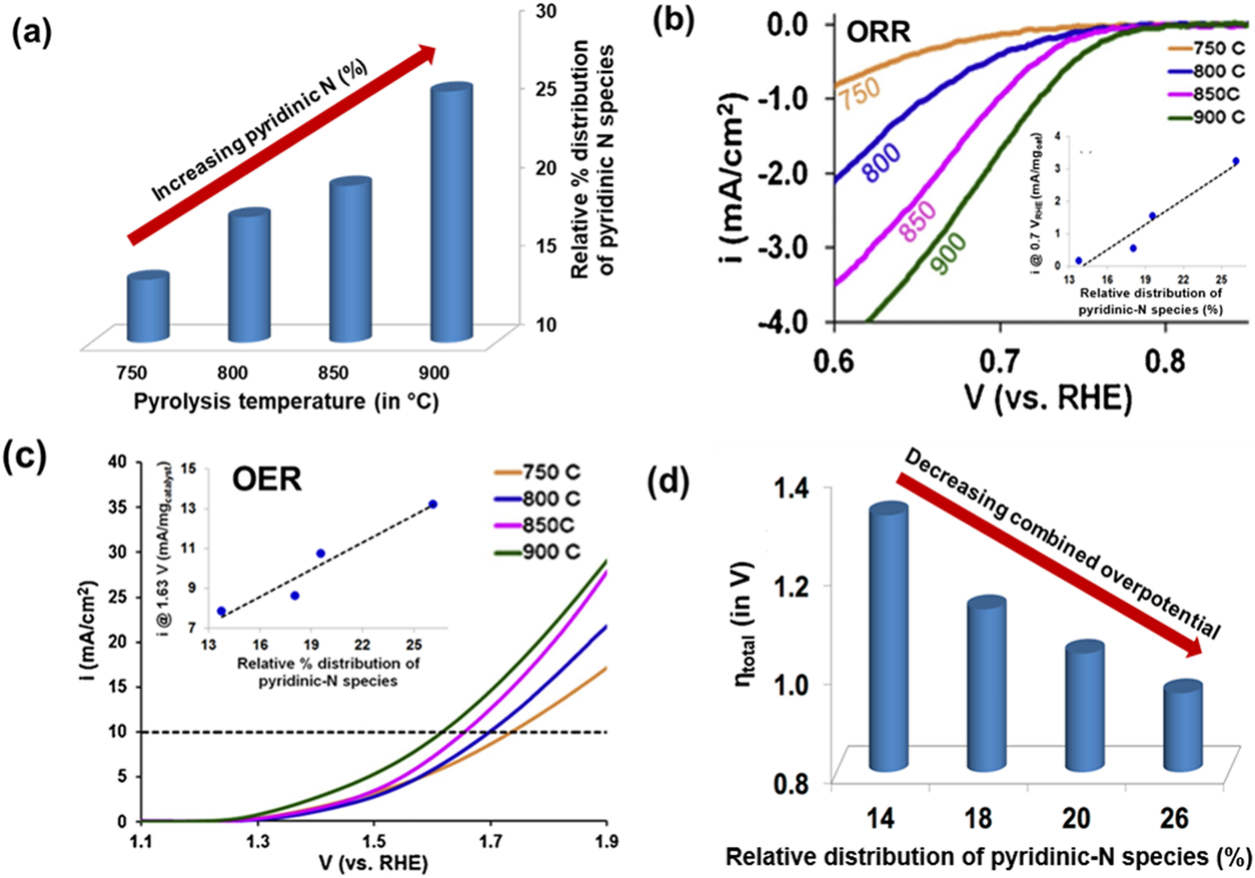
Effect of pyrolysis temperature on (a) pyridinic nitrogen content. (b) ORR activity [inset: specific kinetic current @ 0.7 V vs relative pyridinic-N content]. (c) OER activity [inset: current density @1.63 V vs relative pyridinic-N content]. (d) Correlation of pyridinic-N with combined overpotential for ORR and OER (bifunctionality) [Adapted with permission from ref 35 Copyright 2018 Elsevier].

The ORR and OER activity of these catalysts was tested in an RDE half-cell setup. Figure 7b,c shows the ORR and OER polarization curves for CN_*x*_ catalysts pyrolyzed at different temperatures. Both OER and ORR activity increased with increasing pyrolysis temperatures. The specific current densities correlated almost linearly with relative pyridinic-N content (shown as insets of Figure 7b,c). The highest activity was observed for the CN_*x*_ catalyst with the highest relative composition of pyridinic-N, pyrolyzed at 900 °C, while CN_*x*_ pyrolyzed at 750 °C with the lowest relative composition of pyridinic-N demonstrated the least activity for both OER and ORR. The catalysts also had nearly identical Tafel slopes for ORR, an indicator of a similar rate-determining step for ORR.

The total overpotential (measure of bifunctionality) of CN_*x*_ catalysts also correlated well with the relative distribution of pyridinic-N, where overpotential requirements decreased with an increasing pyridinic-N content (Figure 7d). This indicated that the increase in the pyridinic-N content at higher pyrolysis temperatures improved the bifunctional activity of CN_*x*_. However, for developing a robust catalyst, it was important to evaluate the stability and corrosion resistance of CN_*x*_ under acidic conditions. This is discussed in the next section.

### 2.5 Carbon Corrosion Resistance and Stability of CN_*x*_ Catalysts

Carbon materials often show corrosion tendency in acidic medium. Graphitic materials usually contain oxygen functional groups even at ambient conditions. If carbon corrosion takes place, an increase in the oxygen functionalized groups is observed. Vulcan carbon, a commercial carbon material, has been reported to oxidize and undergo quinone/hydroquinone formation.^100,101^ The degree of carbon corrosion can be determined by monitoring the quinone/hydroquinone redox couple (∼0.6 V) as shown in Figure 8a.^102^ To test the carbon corrosion resistance, intermittent CVs were conducted with chronoamperometric holds for 48 h in an RDE experiment and evolution of quinone to hydroquinone was observed for Vulcan carbon (Figure 8b) and CN_*x*_ (Figure 8c). It can be clearly seen that for Vulcan carbon, the quinone/hydroquinone peak at 0.6 V is visibly intense and increases drastically as the duration of hold potential increases. Contrastingly, CN_*x*_ has a significantly diminished peak, and its intensity did not increase with potential hold duration. These results underscore the excellent carbon corrosion resistance of CN_*x*_ catalysts in acidic environments, making CN_*x*_ suitable for ORR applications.^102^

**Figure 8:**
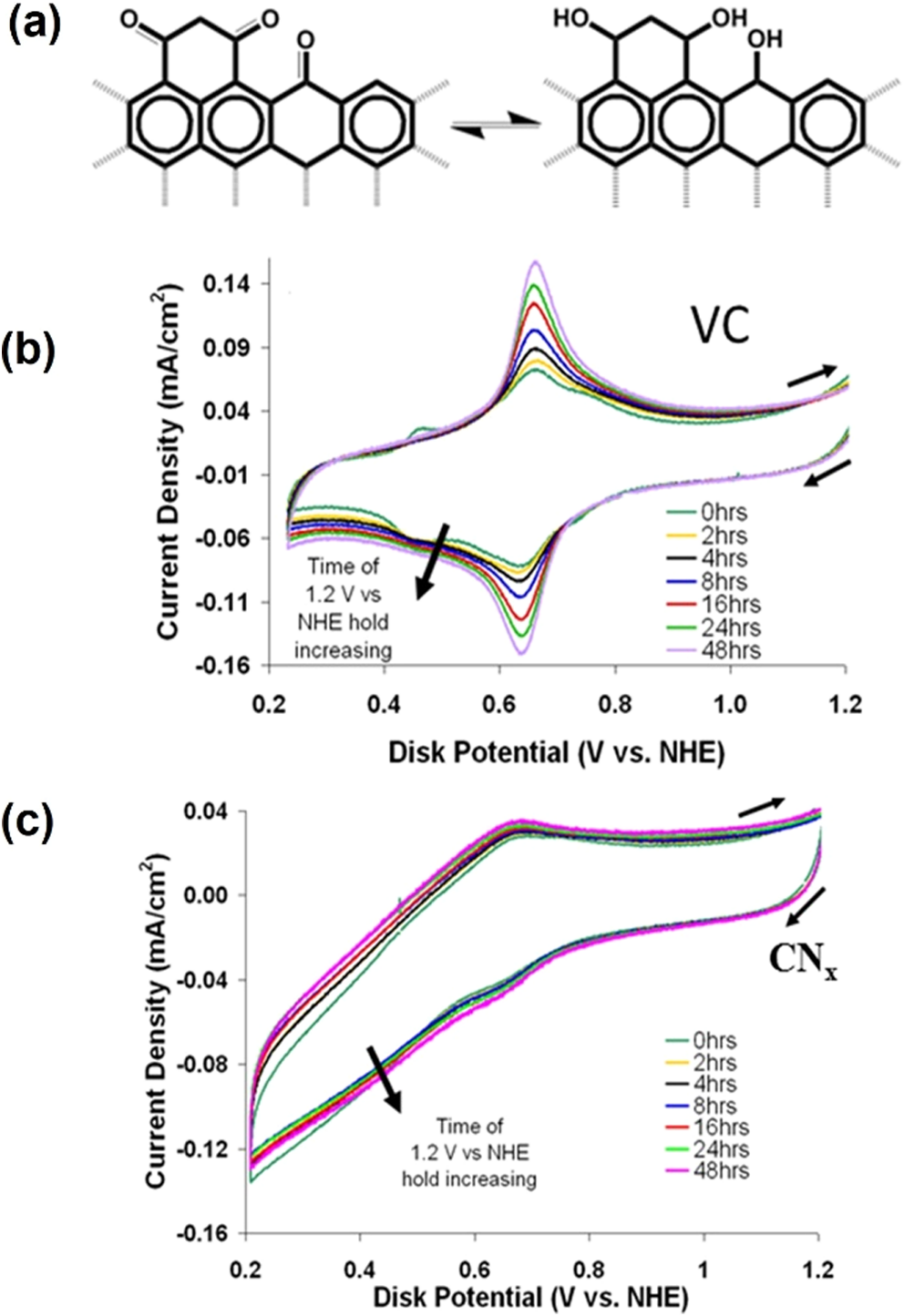
(a) Electrochemically active hydroquinone (right)-quinone (left) reduction–oxidation couple on graphite edge. Evolution of the hydroquinone/quinone species on (b) commercial Vulcan carbon (VC) and (c) CN_*x*_. The CVs were taken after 0, 2, 4, 8, 16, 24, 48 h with 1.2 V vs NHE potential hold [reprinted with permission from ref 102].

The stability of CN_*x*_ for ORR was compared with that of metal-based FeNC via accelerated durability tests (1000 cycles) in the RDE setup. Although CN_*x*_ showed a higher initial activity loss after 100 cycles, it attained a pseudosteady state with no subsequent ORR activity loss between 100 and 1000 cycles. However, FeNC exhibited continued deterioration in ORR activity even up to 1000 cycles. The stability tests were also conducted under fuel cell operation conditions (100 h potential hold at 0.5 V) using an Arbin fuel cell test stand (where the cathode was CN_*x*_). The polarization curves indicated that the loss of activity for CN_*x*_ was considerably lower compared to metal-based catalysts such as FeNC, which showed a dramatic loss of activity after 100 h of potential holding. These results reinforce the excellent stability characteristics of CN_*x*_ catalysts under realistic fuel cell operation conditions.^62^

For the OER, the tolerance to carbon corrosion resistance becomes even more critical as the reaction occurs under highly oxidizing environments/potentials. Under such conditions, carbon can oxidize and undergo oxidation to CO_2_.^103^ The carbon corrosion resistance of CN_*x*_ during OER was examined by testing the effluent gas stream for the presence of CO_2_ using online mass spectrometry. The CO_2_ (*m*/*z* = 44) and O_2_ signals (*m*/*z* = 32) were monitored before (open circuit), during (1 mA/cm^2^ current), and after (open circuit) application of constant current (1 mA/cm^2^), as shown in Figure 9a. An increase in the O_2_ signal and no change in the CO_2_ signal attested to the carbon corrosion resistance of CN_*x*_ and also confirmed that the observed OER current was due to the evolution of oxygen gas and not carbon corrosion. In order to evaluate the long-term stability of CN_*x*_ for the OER, accelerated stability tests were conducted. Current densities as well as the electrochemically active surface areas were measured at different OER cycles. The current densities initially decreased slightly but remained constant in subsequent cycles (Figure 9b). The electrochemically active surface area almost remained unchanged over 100 OER cycles (Figure 9c). These results demonstrated the stability of CN_*x*_ under OER conditions. The corrosion resistance of CN_*x*_ under both ORR and OER conditions further attests to its potential as a bifunctional catalyst for URFC applications.

**Figure 9:**
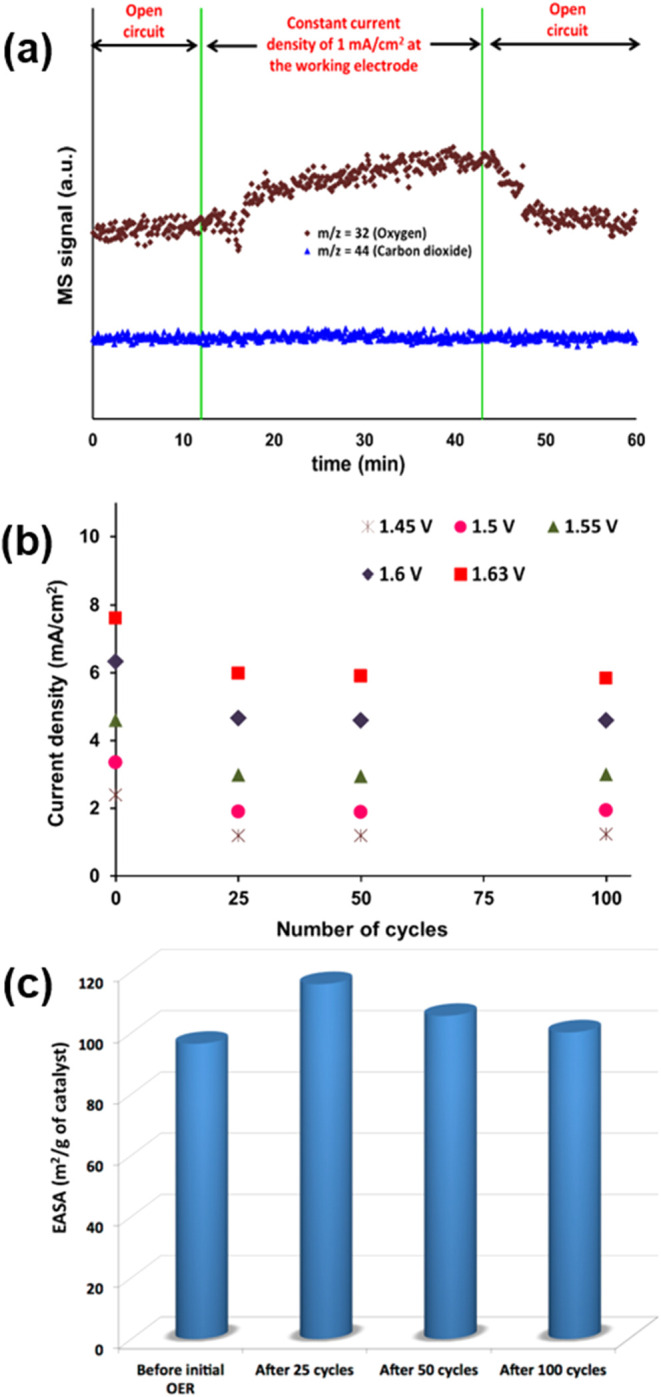
(a) Carbon corrosion test under OER conditions using mass spectrometry: spectra of *m*/*z* = 32 (O_2_) and *m*/*z* = 44 (CO_2_) in exit gas stream. (b) Accelerated durability test for OER. (c) Electrochemically active surface area after various number of cycles in the accelerated durability test for OER. [Reproduced with permission from ref 35 Copyright 2018 Elsevier].

## 3 CN_*x*_ as Catalysts for Electrochemical Halogen Production

Owing to the excellent ORR/OER activity, stability, and poisoning resistance of CN_*x*_ catalysts even in harsh reaction environments, the Ozkan group also studied the performance of CN_*x*_ catalysts for the production of halogens. Halogen production is one of the most energy-intensive processes in the chemical industry.^104^ This section illustrates the application of CN_*x*_ for the energy-efficient production of bromine and chlorine through oxygen-depolarized cathode (ODC) technology.

### 3.1 CN_*x*_ Catalysts for Electrochemical Bromine Production Using ODC Technology

Bromine occurs in nature as bromide in seawater and natural brine deposits. Bromine is mainly used in the production of flame retardants, drilling fluids, drugs, disinfectants for water treatment, and pesticides.^105,106^ Bromine is primarily produced by oxidation of bromide from concentrated brines or seawater or hydrobromic acid, using chlorine gas as a common oxidizing agent, followed by stripping using air or steam. The reaction in bromine production is represented below:

6 $$2{\mathrm{Br}}^{-}+{\mathrm{Cl}}_{2}\to {\mathrm{Br}}_{2}+{2\mathrm{Cl}}^{-}$$

Bromide from anthropogenic activities can enter water bodies and can lead to the formation of reactive and carcinogenic species.^107^ Presence of bromide and iodide in the water disinfected with chlorine increases the cytotoxicity and genotoxicity of the water.^108^ Thus, the bromine evolution reaction (BER) is also relevant in mitigating low-concentration halides from water streams.

The use of highly corrosive and toxic Cl_2_ as an oxidant in traditional bromine production necessitates a more environmentally friendly process for the production of bromine. Moreover, this process can only be economically feasible when bromide concentrations >2.5 g/L (∼30 mM), which makes it unsuitable for streams with low bromide concentrations, such as wastewater.^109^ Advanced technologies such as oxidation using hydrogen peroxide or ozone,^110^ photocatalysis^105^ and electrocatalysis^111^ offer greener alternatives for bromine production. Electrochemical bromine evolution is also relevant in bromine-based redox flow batteries (RFBs), where BER occurs at the anode while hydrogen evolution reaction (HER) (in Hydrogen–Bromine RFBs) or zinc deposition (in Zinc–Bromine RFBs) occurs at the cathode.^112,113^ This section would primarily focus on the electrochemical bromine production performance of CN_*x*_ catalysts.

Electrochemical bromine production conventionally involves the electrolysis of HBr, with BER on the anode and HER on the cathode. However, this process is energy-intensive. Oxygen-depolarized cathode (ODC) technology has emerged as an alternative to reduce energy consumption associated with traditional electrolysis-based production of halogens. In this technology, HER at the cathode is replaced by ORR, which may lead to significant energy savings of up to 30%.^114^ Figure 10 shows a comparison of the redox reactions and thermodynamic cell potential requirements for the traditional process and the ODC process. Clearly, the thermodynamic potential requirements of the ODC process are much lower compared to the traditional process, which translates to a lower energy consumption. In the ODC process, the cathode should not only be active for ORR but also have tolerance to deactivation by crossover of halides from the anode compartment. State-of-the-art ORR catalysts such as Pt/C suffer from deactivation due to halide poisoning, making them unsuitable for ODC applications.^53,115^ CN_*x*_ is active for the ORR and shows excellent tolerance to bromide poisoning^33,115^ (Figure 11), making it suitable for ODC-based halogen production. Moreover, the carbon corrosion resistance of CN_*x*_ confirms its stability under oxidizing potentials in acidic conditions.^35^

**Figure 10:**
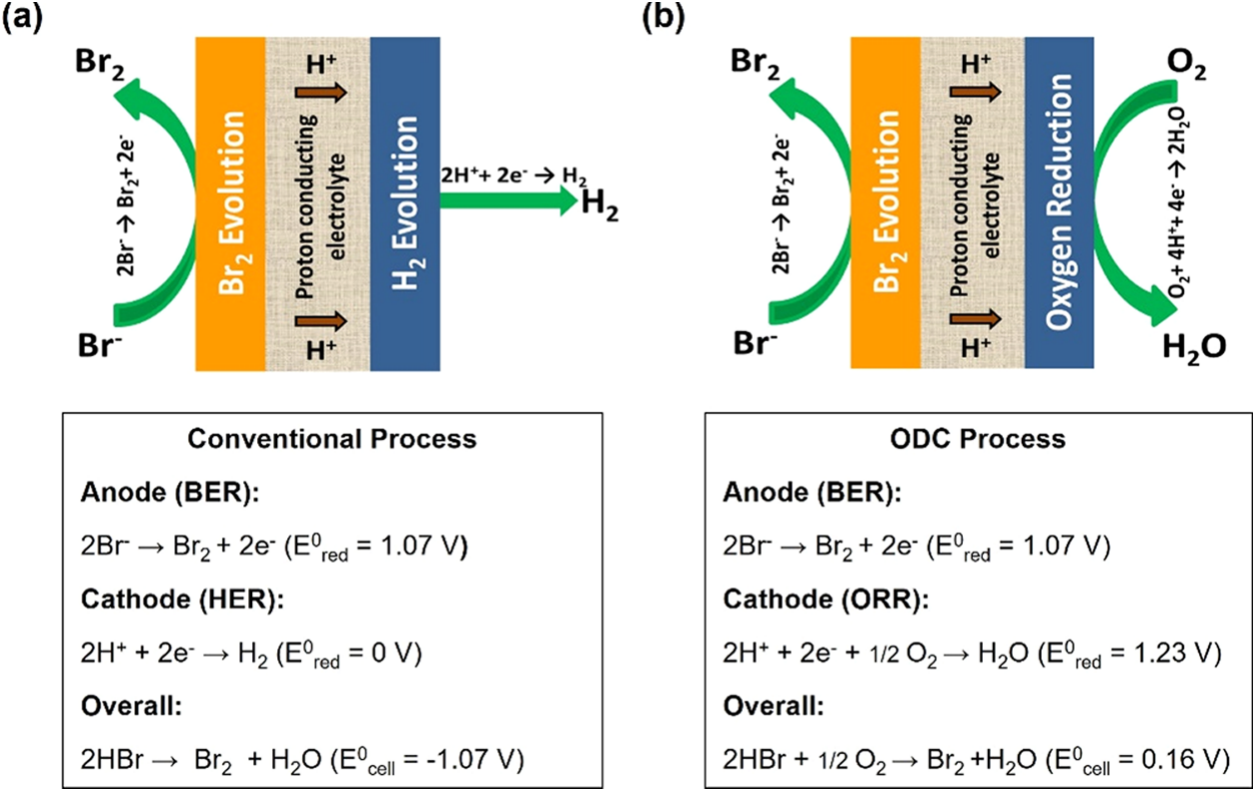
(a) Conventional electrolysis process for electrochemical bromine production. (b) ODC process for electrochemical bromine production [Adapted with permission from ref 116 Copyright 2020 Royal Society of Chemistry].

**Figure 11:**
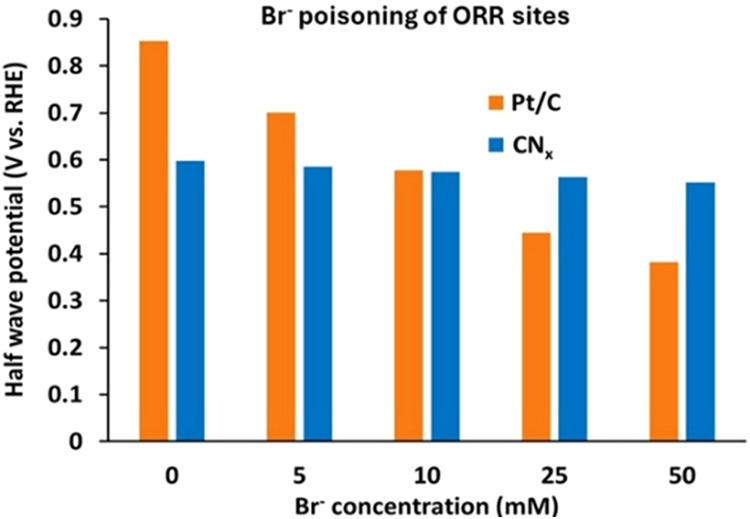
Bromide poisoning tolerance of CN_*x*_ and Pt/C for ORR (reproduced from ref 115 Copyright 2019, open access ETD is published by The Ohio State University and OhioLINK).

### 3.2 CN_*x*_ Materials as Highly Active Electrocatalysts for Bromine Evolution Reaction (BER)

BER via electrochemical oxidation of bromides has been extensively studied using Pt electrodes^117–119^ and graphite electrodes.^120–122^ Similarly, graphite is widely used as an electrode in the chlor-alkali process.^123^ However, graphite requires higher overpotential, and it is unstable due to the electrochemical oxidation of carbon to CO_2_.^123^ Owing to the high corrosivity and oxidizing ability of halogens, using a corrosion-resistant electrode is essential. Thus, precious-metal-based electrodes (most commonly Pt, Ir, and Ru) are heavily used in the halogen production/halide oxidation reactions.^123–126^ Due to the high cost and scarcity of precious metals, much of the research has shifted toward developing corrosion-resistant carbon-based, precious-metal-free electrodes for halogen production.^112^ Co-based spinel oxides, tungsten-oxynitride (WON) nanofibers on graphite felt, titanium nitride (TiN) hollow spheres, Ni/NiO heterostructures, and Sn nanoparticles supported on carbon nanofibers are some of the recently reported nonprecious-metal-based electrodes for the Br^–^/Br_2_ redox reaction in Zn-bromine batteries.^127–131^ Carbon-based materials have advantages such as low cost, intrinsically superior electrical conductivity, high surface area, and chemical resistance to bromine under acidic conditions.^112^ Heteroatom doping of carbon materials could significantly enhance BER activity. CN_*x*_-type materials have been reported as promising electrodes for Zn-bromine flow batteries in the past few years.^132,133^ However, harnessing their potential for bromine production via the ODC technology and probing the nature of BER active sites in CN_*x*_-type catalysts are still ongoing research areas. This section discusses the studies by Ozkan and co-workers on CN_*x*_ as a highly corrosion-resistant electrocatalyst for BER in halogen production.^109,134^

Anodic polarization curves at varying bromide concentrations were collected to assess the BER activity of CN_*x*_, as depicted in Figure 12a. BER activity on CN_*x*_ started at bromide ion concentration as low as 5 mM, as opposed to a minimum of 30 mM that is required for production using chlorine gas as oxidant.^109^ As expected, the activity increased with an increasing bromide concentration as observed by increasing current densities. CN_*x*_ also showed a remarkable selectivity of >99% for BER over competing OER in RRDE experiments. The BER activity of CN_*x*_ was also compared with that of commercial VC and commercial 10% Pt/C (inset of Figure 12a). Vulcan carbon was least active for BER, while CN_*x*_ exhibited the highest BER activity, even surpassing commercial 10% Pt/C. CN_*x*_ also exhibited high stability for up to 1000 cycles of BER tests performed at 25 mM bromide ion concentration, as depicted in Figure 12b. Current densities at 1.2 and 1.3 V remained unchanged throughout 1000 cycles (the inset of Figure 12b). Moreover, BER current densities were compared in the presence of both bromide and chloride ions in solution. Potential hold experiments showed no significant change in BER currents up to 1.4 V (Figure 12c), indicating negligible interference of chlorine evolution, and confirmed the highly selective oxidation of bromide ions in the solution.^115^ This is crucial from the standpoint of selective recovery of bromine from brine streams and seawater, as these streams contain chloride ions in addition to bromide ions.

**Figure 12:**
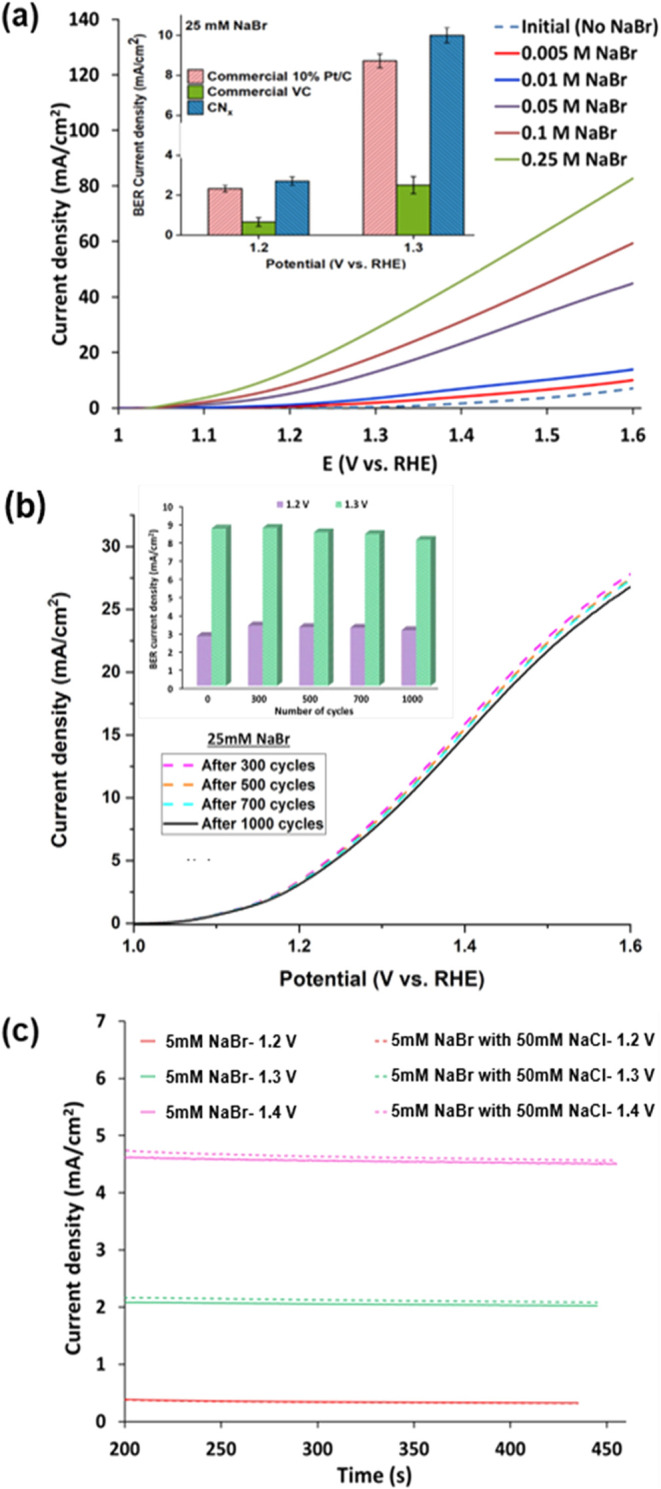
(a) Anodic BER polarization curves at varying bromide ion concentration [inset: comparison of BER current densities for 10% Pt/C, Vulcan carbon (VC), and CN_*x*_]. (b) BER accelerated durability tests [inset: BER current densities after 300, 500, 700, 1000 cycles at 1.2 and 1.3 V] [Adapted with permission from ref 109 Copyright 2022 Elsevier]. (c) BER current densities (for CN*_x_*) in the presence of chloride ions [Adapted from ref 115 Copyright 2019, open access ETD is published by The Ohio State University and OhioLINK].

These results demonstrated that CN*_x_* could function as both an effective anode for BER and a cathode for ORR in an ODC-based halogen production. This enables the development of a “symmetrical” BER cell, where CN_*x*_ would be utilized as both anode as well as cathode, as shown in Figure 13, which could enhance the scale-up and economic potential of ODC technologies. The practical limitations would primarily stem from the corrosive nature of bromine,^135^ which could pose structural integrity challenges with various ODC cell parts.

**Figure 13:**
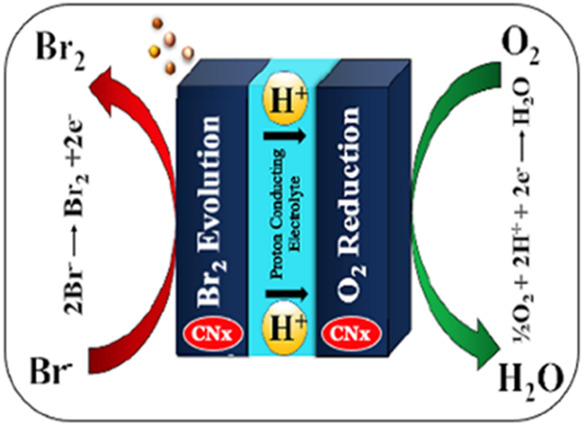
Symmetrical cell for ODC technology-based bromine production where CN_*x*_ serves as both anode and cathode.

### 3.3 CN_*x*_ Catalysts for Electrochemical Chlorine Production via ODC Technology

Chlorine is used in the manufacturing of polycarbonates, epoxy resins, chlorinated intermediates, the chlorohydrin process for propylene oxide production, and in water treatment.^123^ Commercial chlorine production mainly occurs via the chlor-alkali process in a diaphragm or membrane cell.^114,123^ It is estimated that one ton of Cl_2_ needs 2.1–3.0 MWh of electricity, making the process highly energy-intensive.^136^

Other traditional processes, such as electrolysis of HCl, have lower energy consumption compared to the chlor-alkali process but pose safety challenges in handling a reducing gas (H_2_) and oxidizing gas (Cl_2_) together. In contrast, ODC-based Cl_2_ production (Figure 14a) could lower the theoretical voltage from −1.36 V to −0.13 V, just by replacing HER with ORR. It also mitigates the challenges of handling hydrogen gas as the product on the cathode is water. However, poisoning due to chloride ions, stability in an acidic environment, as well as the high cost of Pt catalysts for ORR hinder the widespread commercial applications of ODC-based chlorine production.^136^ Rh-based catalysts such as Rh_*x*_S_*y*_ were extensively developed for ODC due to their superior stability and corrosion resistance over Pt/C.^137^ However, the synthesis procedures of rhodium sulfide-based catalysts often involve handling of extremely toxic H_2_S.^53^ Although there have been H_2_S-free procedures for synthesizing Rh_*x*_S_*y*_,^138^ the elevated cost of rhodium is still a significant roadblock.^136^ Non-noble metal catalysts such as polyoxometalate composites and Cu/Fe_2_O_3_-based catalysts have been explored as cost-effective dual-functional chlorine evolution reaction (CER)/ODC catalysts.^136,139,140^ Carbon-based ORR catalysts (such as CN_*x*_) also have significant potential to be employed as ODC catalysts in chlorine production. Figure 14b shows the tolerance to chloride poisoning (half-wave potential in the presence and absence of chloride ions) of various ODC catalysts. Clearly, CN_*x*_ exhibited remarkable chloride tolerance compared to other ODC catalysts such as Pt/C and Rh_*x*_S_*y*_,^53^ making it a suitable candidate as an ODC for chlorine production.

**Figure 14:**
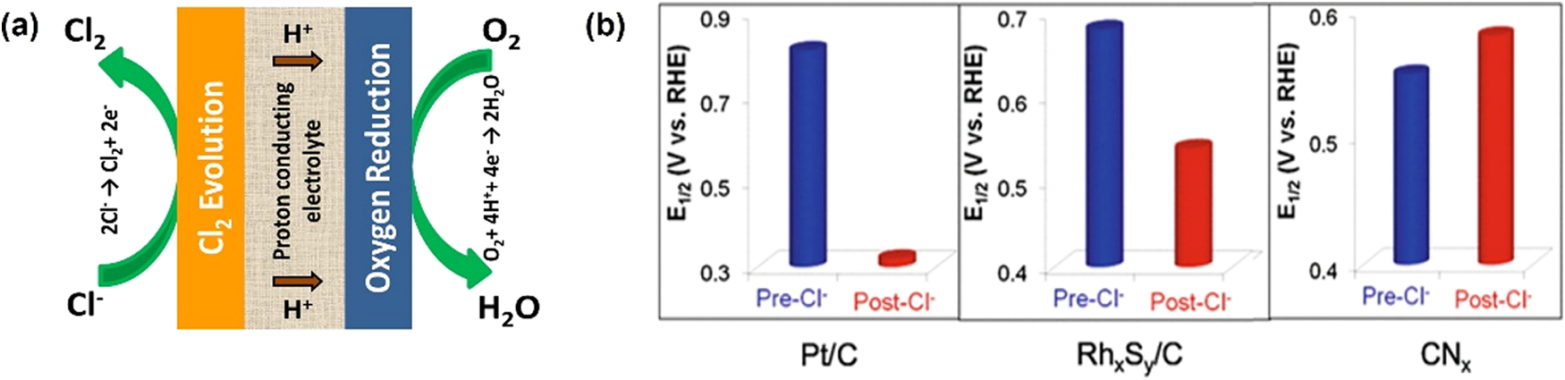
(a) ODC process for chlorine production [Adapted with permission from ref 116 Copyright 2020 Royal Society of Chemistry]. (b) Chloride poisoning tolerance: ORR half-wave potential before and after chloride exposure for various ODC catalysts [Adapted with permission from ref 53 Copyright 2017 Springer Nature].

## 4 Future Perspectives

The results discussed in the preceding sections underscore the potential of CN_*x*_ as a bifunctional ORR/OER electrocatalyst. Moreover, their activity for halogen evolution reaction suggests that these catalysts have significant potential as not just as bifunctional, but also as ‘multifunctional’ electrocatalysts. In this section, some possible future avenues for ‘multifunctional’ electrocatalysis with CN_*x*_ catalysts have been highlighted (Figure 15), specifically focusing on electrosynthesis/electroreduction applications.

**Figure 15:**
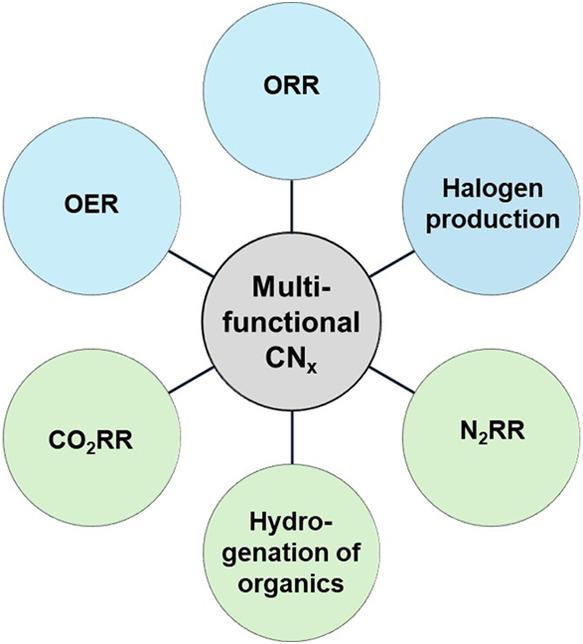
CN_*x*_ as a multifunctional catalyst for various electrocatalytic applications *(circles colored in blue depict the applications discussed in this overview, and circles colored in green represent potential future applications)*.

Efficient and selective electrosynthesis of chemical compounds is one of the upcoming research areas offering advantages such as mild operating conditions, safer processes, and easier integration with renewable energy sources.^141^ However, developing cost-effective electrocatalysts for efficient electrosynthesis of chemicals remains challenging. Electrocatalytic hydrogenation/reduction reactions have been widely explored in the domain of electrosynthesis, as they play a critical role in the production of value-added chemicals,^142,143^ especially for the removal of pollutants, synthesis of essential chemicals, as well as carbon capture and utilization. Hydrogenation processes consume a significant fraction of global energy, with ammonia production alone accounting for ∼1% of global energy supply.^144^ Economically viable electrification of these chemical processes could have a significant impact on enhancing the operational safety (as hydrogen gas is substituted by water/protons), milder reaction conditions, and reduction of carbon emissions by integration with renewable and clean energy sources. Some electroreduction reactions where CN_*x*_ can prove to be a promising and cost-effective alternative are discussed in this section.

Developing an effective, economical CO_2_ electroreduction (CO_2_RR) process is crucial for decarbonization of the chemical industry. Although CO_2_RR has been predominantly explored on metal-based catalysts, there have been some studies to utilize metal-free CN_*x*_ materials for CO_2_RR.^145,146^ Wu et al. demonstrated the CO_2_ reduction reaction (CO_2_RR) to CO using nitrogen-incorporated graphene foam.^147^ N-doped graphene quantum dots also showed considerable CO_2_RR activity toward multicarbon hydrocarbons and oxygenates.^148^ Although these studies highlight promising applications of CN_*x*_ as a metal-free catalyst for CO_2_RR, developing structure–activity correlations for the rational design of tunable and selective catalysts along with a comprehensive mechanistic understanding of CO_2_RR still remains challenging.

Another area of extensive research is nitrogen (N_2_) electroreduction. Due to the exceptional stability of the N–N bond, activation of N_2_ is extremely challenging.^149^ The conventional thermocatalytic Haber-Bosch process for ammonia production is highly energy- and carbon-intensive.^150–152^ It is also not feasible for modular production due to economic feasibility challenges associated with smaller operation scales, often resulting in concentration of production hubs at specific geographical locations and elevating the logistics costs for transportation.^153^ Electrochemical production of ammonia provides a modular production strategy, and its integration with clean energy can significantly reduce the carbon footprint.^153,154^ Both low- and high-temperature N_2_ reduction reactions (N_2_RRs) are garnering significant attention, especially when H_2_O/H^+^ is used as the hydrogen source instead of H_2_.^155–160^ Several studies have recently explored heteroatom-doped carbon catalysts for electrochemical ammonia production.^161–164^ However, challenges arising due to sluggish N_2_RR kinetics at room temperatures, mechanistic insights, and complexities in solution-phase synthesis and reliability of ammonia quantification strategies need to be addressed in future studies involving aqueous phase ammonia production.^165–167^

There has also been an emerging interest in developing catalysts for electrocatalytic hydrogenation of organic molecules, especially for the upgrading of biomass-derived organics.^168^ Electrocatalytic hydrogenation is driven by electricity using protons/water^169^ and eliminates the handling of strong reducing agents or high-pressure hydrogen gas, making the hydrogenation processes safer.^170^ The electrocatalytic hydrogenation (ECH) reactions are predominantly catalyzed by precious-metal-based catalysts^171^ or even pure precious metals,^172^ reducing their economic viability. Recent studies have focused on developing electrocatalysts derived from nonprecious metals^173–175^ or even metal-free catalysts.^176,177^ Grammenos et al. recently reported nitrogen-doped carbon catalysts for effective ECH of maleic acid to succinic acid in an acidic medium.^178^ If CN_*x*_ can catalyze the hydrogenation reactions on the cathode and the OER on the anode, it offers another possibility of a symmetrical cell. Such works have opened new avenues in electrocatalysis research on CN_*x*_ catalysts into the domain of organic electrosynthesis under mild conditions.

## 5 CN_*x*_ as a Multifunctional Catalyst: Challenges

As seen in the previous section, CN_*x*_ materials can catalyze a wide range of electrocatalytic reactions and can be developed as potential cost-effective “multifunctional” electrocatalysts. However, the most crucial challenge for the multifunctional applications of CN_*x*_ catalysts is the determination of the exact nature of the active sites of CN_*x*_ catalysts. Although pyridinic-N sites have been widely recognized as ORR active sites/markers,^66,72^ that may not necessarily be true for electro-hydrogenation and halogen evolution reactions. Moreover, synthesizing CN_*x*_-type catalysts with a precise distribution of active sites is difficult to achieve.^179^ Hence, deciphering the nature of active sites using advancements in theoretical understanding of CN_*x*_-type catalysts (DFT studies/machine learning models) coupled with electrochemical poisoning studies would be essential for multifunctional applications of these catalysts. To further enhance the understanding of these active sites, real-time investigation of dynamic changes in the active sites under applied potentials using in situ/operando characterization studies is important. However, the complexity of the electrocatalytic systems and CN_*x*_-type electrode materials consisting of light elements such as C, N, and O, which exhibit relatively weak scattering and absorption signals in various spectroscopic and microscopic techniques, makes the in situ/operando investigation of these systems difficult.^180^ Additionally, a lack of mechanistic understanding of electrosynthesis-type reactions with a much higher degree of complexity than seemingly simple-looking ORR/OER is another major challenge in the multifunctionality of CN_*x*_. Thus, fundamental studies focusing on expanding the mechanistic understanding of the interplay of various intermediates in complex multistep electro-hydrogenation reactions would be essential for engineering active and selective ‘multifunctional’ CN_*x*_ catalysts.

## 6 Conclusions

The studies described in this overview illustrate the bifunctional ORR and OER activity of precious-metal-free CN_*x*_ for applications in URFCs. Applications of CN_*x*_ for electrochemical production of halogens (chlorine and bromine) via ODC technology were also discussed. Excellent bromine evolution and oxygen reduction activity, combined with ORR poisoning resistance toward bromides, make CN_*x*_ an attractive electrode choice for a potential symmetrical cell for bromine production. Additionally, some potential avenues of future electrocatalysis research for developing CN_*x*_ as a ‘multifunctional’ electrocatalyst are emerging.

## References

[1] Yang Z., Nie H., Chen Xa., Chen X., Huang S. (2013). Recent progress in
doped carbon nanomaterials as effective cathode catalysts for fuel
cell oxygen reduction reaction. J. Power Sources.

[2] Gewirth A. A., Varnell J. A., DiAscro A. M. (2018). Nonprecious
metal catalysts for oxygen
reduction in heterogeneous aqueous systems. Chem. Rev..

[3] Hu C., Dai L. (2016). Carbon-based metal-free catalysts for electrocatalysis
beyond the
ORR. Angew. Chem., Int. Ed..

[4] Dai L. (2017). Carbon-based
catalysts for metal-free electrocatalysis. Curr.
Opin. Electrochem..

[5] Perini L., Durante C., Favaro M., Perazzolo V., Agnoli S., Schneider O., Granozzi G., Gennaro A. (2015). Metal–support
interaction in platinum and palladium nanoparticles loaded on nitrogen-doped
mesoporous carbon for oxygen reduction reaction. ACS Appl. Mater. Interfaces.

[6] Melke J., Peter B., Habereder A., Ziegler J., Fasel C., Nefedov A., Sezen H., Wöll C., Ehrenberg H., Roth C. (2016). Metal–support
interactions
of platinum nanoparticles decorated N-doped carbon nanofibers for
the oxygen reduction reaction. ACS Appl. Mater.
Interfaces.

[7] Nagaiah T. C., Kundu S., Bron M., Muhler M., Schuhmann W. (2010). Nitrogen-doped
carbon nanotubes as a cathode catalyst for the oxygen reduction reaction
in alkaline medium. Electrochem. Commun..

[8] Biddinger E. J., Von Deak D., Ozkan U. S. (2009). Nitrogen-containing carbon nanostructures
as oxygen-reduction catalysts. Top. Catal..

[9] Matter P. H., Wang E., Arias M., Biddinger E. J., Ozkan U. S. (2007). Oxygen reduction reaction activity
and surface properties
of nanostructured nitrogen-containing carbon. J. Mol. Catal. A: Chem..

[10] Matter P. H., Zhang L., Ozkan U. S. (2006). The role
of nanostructure in nitrogen-containing
carbon catalysts for the oxygen reduction reaction. J. Catal..

[11] Matter P. H., Wang E., Ozkan U. S. (2006). Preparation of nanostructured
nitrogen-containing
carbon catalysts for the oxygen reduction reaction from SiO2-and MgO-supported
metal particles. J. Catal..

[12] Matter P. H., Wang E., Arias M., Biddinger E. J., Ozkan U. S. (2006). Oxygen reduction reaction catalysts
prepared from acetonitrile
pyrolysis over alumina-supported metal particles. J. Phys. Chem. B.

[13] Matter P. H., Ozkan U. S. (2006). Non-metal catalysts
for dioxygen reduction in an acidic
electrolyte. Catal. Lett..

[14] Maldonado S., Stevenson K. J. (2005). Influence
of nitrogen doping on oxygen reduction electrocatalysis
at carbon nanofiber electrodes. J. Phys. Chem.
B.

[15] Li X., Liu G., Popov B. N. (2010). Activity and stability of non-precious metal catalysts
for oxygen reduction in acid and alkaline electrolytes. J. Power Sources.

[16] Qu L., Liu Y., Baek J.-B., Dai L. (2010). Nitrogen-doped graphene as efficient
metal-free electrocatalyst for oxygen reduction in fuel cells. ACS Nano.

[17] Biddinger E. J., Von Deak D., Singh D., Marsh H., Tan B., Knapke D. S., Ozkan U. S. (2011). Examination
of catalyst loading effects
on the selectivity of CNx and Pt/VC ORR catalysts using RRDE. J. Electrochem. Soc..

[18] Chen Z., Higgins D., Tao H., Hsu R. S., Chen Z. (2009). Highly active
nitrogen-doped carbon nanotubes for oxygen reduction reaction in fuel
cell applications. J. Phys. Chem. C.

[19] Shao Y., Sui J., Yin G., Gao Y. (2008). Nitrogen-doped carbon nanostructures
and their composites as catalytic materials for proton exchange membrane
fuel cell. Appl. Catal., B.

[20] Zhuang S., Nunna B. B., Mandal D., Lee E. S. (2018). A review of nitrogen-doped
graphene catalysts for proton exchange membrane fuel cells-synthesis,
characterization, and improvement. Nano-Struct.
Nano-Objects.

[21] Wood K. N., O’Hayre R., Pylypenko S. (2014). Recent progress on nitrogen/carbon
structures designed for use in energy and sustainability applications. Energy Environ. Sci..

[22] Deokar G., Jin J., Schwingenschlögl U., Costa P. M. (2022). Chemical vapor deposition-grown
nitrogen-doped graphene’s synthesis, characterization and applications. npj 2D Mater. Appl..

[23] Singh D., Soykal I. I., Tian J., von Deak D., King J., Miller J. T., Ozkan U. S. (2013). In situ characterization
of the growth
of CNx carbon nano-structures as oxygen reduction reaction catalysts. J. Catal..

[24] Matter P. H., Wang E., Millet J.-M. M., Ozkan U. S. (2007). Characterization
of the iron phase in CN x-based oxygen reduction reaction catalysts. J. Phys. Chem. C.

[25] Matter, P. H. ; Biddinger, E. J. ; Ozkan, U. S. Non-Precious Metal Oxygen Reduction Catalysts for PEM Fuel Cells. In Catalysis; RSC Publishing, 2007 10.1039/b602370n.

[26] Mamtani K., Ozkan U. S. (2015). Heteroatom-doped carbon nanostructures as oxygen reduction
reaction catalysts in acidic media: an overview. Catal. Lett..

[27] Jain D., Zhang Q., Hightower J., Gustin V., Asthagiri A., Ozkan U. S. (2019). Changes in Active
Sites on Nitrogen-Doped Carbon Catalysts
Under Oxygen Reduction Reaction: A Combined Post-Reaction Characterization
and DFT Study. ChemCatChem.

[28] Hayashida K., Lu B., Takakusagi S., Nakamura J., Takeyasu K. (2025). Design Principles of
Nitrogen-Doped Carbon Catalysts for Oxygen Reduction Reaction. ChemElectroChem.

[29] Biddinger E. J., Ozkan U. S. (2010). Role of graphitic
edge plane exposure in carbon nanostructures
for oxygen reduction reaction. J. Phys. Chem.
C.

[30] Gustin V., Sohale A., Mehta S., Vennala N., Gunduz S., Khalifa Y., Conroy D., Co A. C., Asthagiri A., Ozkan U. S. (2025). Doping CN x Catalysts with Boron: Structural Changes
and Implications for Oxygen Reduction Reaction Activity. Energy Fuels.

[31] Von
Deak D., Biddinger E. J., Luthman K. A., Ozkan U. S. (2010). The effect of phosphorus
in nitrogen-containing carbon nanostructures on oxygen reduction in
PEM fuel cells. Carbon.

[32] Biddinger E. J., Knapke D. S., von Deak D., Ozkan U. S. (2010). Effect of sulfur
as a growth promoter for CNx nanostructures as PEM and DMFC ORR catalysts. Appl. Catal., B.

[33] Basu D., Vennala N., Sohale A., McFarlane P., Nordlund D., Gunduz S., Co A. C., Asthagiri A., Ozkan U. S. (2025). Effect of electrochemical bromide
doping on the performance
of nitrogen-doped carbon nanostructures for oxygen reduction reaction. Appl. Catal., B.

[34] Jain D., Mamtani K., Gustin V., Gunduz S., Celik G., Waluyo I., Hunt A., Co A. C., Ozkan U. S. (2018). Enhancement
in oxygen reduction reaction activity of nitrogen-doped carbon nanostructures
in acidic media through chloride-ion exposure. ChemElectroChem.

[35] Mamtani K., Jain D., Dogu D., Gustin V., Gunduz S., Co A. C., Ozkan U. S. (2018). Insights
into oxygen reduction reaction
(ORR) and oxygen evolution reaction (OER) active sites for nitrogen-doped
carbon nanostructures (CNx) in acidic media. Appl. Catal., B.

[36] Zhang X., Zhang X., Zhao S., Wang Y. Q., Lin X., Tian Z. Q., Shen P. K., Jiang S. P. (2021). Precursor modulated
active sites of nitrogen doped graphene-based carbon catalysts via
one-step pyrolysis method for the enhanced oxygen reduction reaction. Electrochim. Acta.

[37] Miao H., Li S., Wang Z., Sun S., Kuang M., Liu Z., Yuan J. (2017). Enhancing the pyridinic
N content of Nitrogen-doped graphene and
improving its catalytic activity for oxygen reduction reaction. Int. J. Hydrogen Energy.

[38] Masa J., Xia W., Muhler M., Schuhmann W. (2015). On the role of metals in nitrogen-doped
carbon electrocatalysts for oxygen reduction. Angew. Chem., Int. Ed..

[39] Masa J., Zhao A., Xia W., Sun Z., Mei B., Muhler M., Schuhmann W. (2013). Trace metal
residues promote the
activity of supposedly metal-free nitrogen-modified carbon catalysts
for the oxygen reduction reaction. Electrochem.
Commun..

[40] Lefèvre M., Proietti E., Jaouen F., Dodelet J.-P. (2009). Iron-based catalysts
with improved oxygen reduction activity in polymer electrolyte fuel
cells. Science.

[41] Kramm U. I., Lefèvre M., Larouche N., Schmeisser D., Dodelet J.-P. (2014). Correlations between
mass activity and physicochemical
properties of Fe/N/C catalysts for the ORR in PEM fuel cell via 57Fe
Mossbauer spectroscopy and other techniques. J. Am. Chem. Soc..

[42] Gupta S., Tryk D., Bae I., Aldred W., Yeager E. (1989). Heat-treated
polyacrylonitrile-based catalysts for oxygen electroreduction. J. Appl. Electrochem..

[43] Gupta S., Fierro C., Yeager E. (1991). The effects
of cyanide on the electrochemical
properties of transition metal macrocycles for oxygen reduction in
alkaline solutions. J. Electroanal. Chem. Interfacial
Electrochem..

[44] Wu G., More K. L., Johnston C. M., Zelenay P. (2011). High-performance electrocatalysts
for oxygen reduction derived from polyaniline, iron, and cobalt. Science.

[45] Wu G., Zelenay P. (2013). Nanostructured nonprecious
metal catalysts for oxygen
reduction reaction. Acc. Chem. Res..

[46] Chung H. T., Cullen D. A., Higgins D., Sneed B. T., Holby E. F., More K. L., Zelenay P. (2017). Direct atomic-level
insight into
the active sites of a high-performance PGM-free ORR catalyst. Science.

[47] Mamtani K., Jain D., Co A. C., Ozkan U. S. (2017). Nitrogen-coordinated
iron– carbon as efficient bifunctional electrocatalysts for
the oxygen reduction and oxygen evolution reactions in acidic media. Energy Fuels.

[48] Yu D., Zhang Q., Dai L. (2010). Highly efficient
metal-free growth
of nitrogen-doped single-walled carbon nanotubes on plasma-etched
substrates for oxygen reduction. J. Am. Chem.
Soc..

[49] Von
Deak D., Singh D., Biddinger E. J., King J. C., Bayram B., Miller J. T., Ozkan U. S. (2012). Investigation of sulfur poisoning
of CNx oxygen reduction catalysts for PEM fuel cells. J. Catal..

[50] von
Deak D., Singh D., King J. C., Ozkan U. S. (2012). Use of carbon monoxide
and cyanide to probe the active sites on nitrogen-doped carbon catalysts
for oxygen reduction. Appl. Catal., B.

[51] Singh D., Mamtani K., Bruening C. R., Miller J. T., Ozkan U. S. (2014). Use of
H2S to probe the active sites in FeNC catalysts for the oxygen reduction
reaction (ORR) in acidic media. ACS Catal..

[52] Zhang Q., Mamtani K., Jain D., Ozkan U., Asthagiri A. (2016). CO poisoning
effects on FeNC and CN x ORR catalysts: A combined experimental–computational
study. J. Phys. Chem. C.

[53] Mamtani K., Jain D., Co A. C., Ozkan U. S. (2017). Investigation of
chloride poisoning resistance for nitrogen-doped carbon nanostructures
as oxygen depolarized cathode catalysts in acidic media. Catal. Lett..

[54] Gong K., Du F., Xia Z., Durstock M., Dai L. (2009). Nitrogen-doped carbon
nanotube arrays with high electrocatalytic activity for oxygen reduction. Science.

[55] Wang Q., Zhou Z.-Y., Lai Y.-J., You Y., Liu J.-G., Wu X.-L., Terefe E., Chen C., Song L., Rauf M. (2014). Phenylenediamine-based
FeN x/C catalyst with high activity
for oxygen reduction in acid medium and its active-site probing. J. Am. Chem. Soc..

[56] Dai L., Chang D. W., Baek J. B., Lu W. (2012). Carbon nanomaterials
for advanced energy conversion and storage. Small.

[57] Wu K. H., Wang D. W., Su D. S., Gentle I. R. (2015). A discussion on
the activity origin in metal-free nitrogen-doped carbons for oxygen
reduction reaction and their mechanisms. ChemSusChem.

[58] Cherif M., Dodelet J.-P., Zhang G., Glibin V. P., Sun S., Vidal F. (2021). Non-PGM electrocatalysts for PEM fuel cells: a DFT study on the effects
of fluorination of FeNx-doped and N-doped carbon catalysts. Molecules.

[59] Qi Z., He C., Kaufman A. (2002). Effect of CO in the anode fuel on
the performance of
PEM fuel cell cathode. J. Power Sources.

[60] Biddinger E. J., Ozkan U. S. (2007). Methanol tolerance of CN x oxygen reduction catalysts. Top. Catal..

[61] Mamtani K., Singh D., Tian J., Millet J.-M. M., Miller J. T., Co A. C., Ozkan U. S. (2016). Evolution of N-coordinated
iron–carbon
(FeNC) catalysts and their oxygen reduction (ORR) performance in acidic
media at various stages of catalyst synthesis: an attempt at benchmarking. Catal. Lett..

[62] Singh D., Tian J., Mamtani K., King J., Miller J. T., Ozkan U. S. (2014). A comparison of N-containing carbon
nanostructures
(CNx) and N-coordinated iron–carbon catalysts (FeNC) for the
oxygen reduction reaction in acidic media. J.
Catal..

[63] Mamtani K., Singh D., Dogu D., Jain D., Millet J.-M. M., Ozkan U. S. (2018). Effect of acid-washing on the nature of bulk characteristics
of nitrogen-doped carbon nanostructures as oxygen reduction reaction
electrocatalysts in acidic media. Energy Fuels.

[64] Wang L., Xiao J., Mao Q., Cai C., Zhong Q., Liu C., Liu M. (2025). Fe3O4 encapsulated in hierarchically porous nitrogen-doped
graphitic carbon layers for efficient oxygen reduction reaction: Enhanced
intrinsic activity via directional interfacial charge transfer. J. Colloid Interface Sci..

[65] Guo D., Shibuya R., Akiba C., Saji S., Kondo T., Nakamura J. (2016). Active sites of nitrogen-doped
carbon materials for
oxygen reduction reaction clarified using model catalysts. Science.

[66] Mamtani K., Jain D., Zemlyanov D., Celik G., Luthman J., Renkes G., Co A. C., Ozkan U. S. (2016). Probing the oxygen
reduction reaction active sites over nitrogen-doped carbon nanostructures
(CN x) in acidic media using phosphate anion. ACS Catal..

[67] Xing T., Zheng Y., Li L. H., Cowie B. C., Gunzelmann D., Qiao S. Z., Huang S., Chen Y. (2014). Observation
of active
sites for oxygen reduction reaction on nitrogen-doped multilayer graphene. ACS Nano.

[68] Rao C. V., Cabrera C. R., Ishikawa Y. (2010). In search
of the active site in nitrogen-doped
carbon nanotube electrodes for the oxygen reduction reaction. J. Phys. Chem. Lett..

[69] Quílez-Bermejo J., Melle-Franco M., San-Fabián E., Morallón E., Cazorla-Amorós D. (2019). Towards understanding
the active
sites for the ORR in N-doped carbon materials through fine-tuning
of nitrogen functionalities: an experimental and computational approach. J. Mater. Chem. A.

[70] Wang N., Lu B., Li L., Niu W., Tang Z., Kang X., Chen S. (2018). Graphitic nitrogen
is responsible for oxygen electroreduction on
nitrogen-doped carbons in alkaline electrolytes: insights from activity
attenuation studies and theoretical calculations. ACS Catal..

[71] Behan J. A., Iannaci A., Dominguez C., Stamatin S. N., Hoque M. K., Vasconcelos J. M., Perova T. S., Colavita P. E. (2019). Electrocatalysis
of N-doped carbons in the oxygen reduction reaction as a function
of pH: N-sites and scaffold effects. Carbon.

[72] Wang T., Chen Z.-X., Chen Y.-G., Yang L.-J., Yang X.-D., Ye J.-Y., Xia H.-P., Zhou Z.-Y., Sun S.-G. (2018). Identifying
the active site of N-doped graphene for oxygen reduction by selective
chemical modification. ACS Energy Lett..

[73] Rao A., Gustin V., Hightower J., Gunduz S., Basu D., Khalifa Y., Sohale A., Co A. C., Asthagiri A., Ozkan U. S. (2025). CO2 Poisoning of CN x Catalysts for the Oxygen Reduction
Reaction. J. Phys. Chem. C.

[74] Quílez-Bermejo J., Morallón E., Cazorla-Amorós D. (2020). Metal-free heteroatom-doped
carbon-based catalysts for ORR: A critical assessment about the role
of heteroatoms. Carbon.

[75] Rana M., Mondal S., Sahoo L., Chatterjee K., Karthik P. E., Gautam U. K. (2018). Emerging materials
in heterogeneous
electrocatalysis involving oxygen for energy harvesting. ACS Appl. Mater. Interfaces.

[76] Zhu J., Huang Y., Mei W., Zhao C., Zhang C., Zhang J., Amiinu I. S., Mu S. (2019). Effects of intrinsic
pentagon defects on electrochemical reactivity of carbon nanomaterials. Angew. Chem., Int. Ed..

[77] Zhu X., Shao Y., Xia D., Wei Y., Li Z., Liu W., Wang N., Wu Q., Ding F., Li J. (2025). When Graphitic Nitrogen
Meets Pentagons: Selective Construction and
Spectroscopic Evidence for Improved Four-Electron Oxygen Reduction
Electrocatalysis. Adv. Mater..

[78] Jia Y., Zhang L., Zhuang L., Liu H., Yan X., Wang X., Liu J., Wang J., Zheng Y., Xiao Z. (2019). Identification of active sites for acidic oxygen reduction
on carbon catalysts with and without nitrogen doping. Nat. Catal..

[79] Duan N., Wang J., Wang R., Han G., Wu X., Liu Y., Li B. (2025). Progress and Perspective of Noble-Metal-Free
Bifunctional
Oxygen Electrocatalysts for Zinc-Air Batteries. Adv. Sustainable Syst..

[80] Liu H., Xiong R., Ma S., Wang R., Liu Z., Yao T., Song B. (2025). Recent advances in noble-metal-free bifunctional oxygen
electrode catalysts. Energy Adv..

[81] Liu H., Liu Q., Wang Y., Wang Y., Chou S., Hu Z., Zhang Z. (2022). Bifunctional
carbon-based cathode catalysts for zinc-air battery:
A review. Chin. Chem. Lett..

[82] Asefa T. (2016). Metal-free
and noble metal-free heteroatom-doped nanostructured carbons as prospective
sustainable electrocatalysts. Acc. Chem. Res..

[83] Hassan Q., Viktor P., Al-Musawi T. J., Ali B. M., Algburi S., Alzoubi H. M., Al-Jiboory A. K., Sameen A. Z., Salman H. M., Jaszczur M. (2024). The renewable energy role in the global energy Transformations. Renewable Energy Focus.

[84] Mlilo N., Brown J., Ahfock T. (2021). Impact of
intermittent renewable
energy generation penetration on the power system networks–A
review. Technol. Econ. Smart Grids Sustainable
Energy.

[85] Wang Y., Leung D. Y., Xuan J., Wang H. (2016). A review on unitized
regenerative fuel cell technologies, part-A: Unitized regenerative
proton exchange membrane fuel cells. Renewable
Sustainable Energy Rev..

[86] Kiani M., Zhao Y., Zhang R. (2025). Proton exchange membrane
fuel cells:
recent developments and future perspectives. Chem. Commun..

[87] Wang Y., Pang Y., Xu H., Martinez A., Chen K. S. (2022). PEM Fuel
cell and electrolysis cell technologies and hydrogen infrastructure
development–a review. Energy Environ.
Sci..

[88] Abbas M. A., Bang J. H. (2015). Rising again: opportunities and challenges for platinum-free
electrocatalysts. Chem. Mater..

[89] Spöri C., Briois P., Nong H. N., Reier T., Billard A., Kühl S., Teschner D., Strasser P. (2019). Experimental activity
descriptors for iridium-based catalysts for the electrochemical oxygen
evolution reaction (OER). ACS Catal..

[90] Mamtani, K. ; Ozkan, U. S. Nitrogen-Doped Carbon Nanostructures as Oxygen Reduction Reaction (ORR) and Oxygen Evolution Reaction (OER) Electrocatalysts in Acidic Media. In Handbook of Graphene; Wiley, 2019 10.1002/9781119468455.ch80.

[91] Filimonenkov I. S., Bouillet C., Kéranguéven G., Simonov P. A., Tsirlina G. A., Savinova E. R. (2019). Carbon materials as additives to
the OER catalysts: RRDE study of carbon corrosion at high anodic potentials. Electrochim. Acta.

[92] Kim I. G., Nah I. W., Oh I.-H., Park S. (2017). Crumpled rGO-supported
Pt-Ir bifunctional catalyst prepared by spray pyrolysis for unitized
regenerative fuel cells. J. Power Sources.

[93] Zhu S., Liu Y., Gong Y., Sun Y., Chen K., Liu Y., Liu W., Xia T., Zheng Q., Gao H. (2024). Boosting bifunctional
catalysis by integrating active faceted intermetallic nanocrystals
and strained Pt–Ir functional shells. Small.

[94] Ng J. W. D., Tang M., Jaramillo T. F. (2014). A carbon-free,
precious-metal-free,
high-performance O 2 electrode for regenerative fuel cells and metal–air
batteries. Energy Environ. Sci..

[95] Bhatt M. D., Lee J. Y. (2020). Advancement of platinum
(Pt)-free (non-Pt precious
metals) and/or metal-free (non-precious-metals) electrocatalysts in
energy applications: A review and perspectives. Energy Fuels.

[96] To J. W. F., Ng J. W. D., Siahrostami S., Koh A. L., Lee Y., Chen Z., Fong K. D., Chen S., He J., Bae W.-G. (2017). High-performance
oxygen reduction and evolution carbon
catalysis: From mechanistic studies to device integration. Nano Res..

[97] Yang G., Zhu J., Yuan P., Hu Y., Qu G., Lu B.-A., Xue X., Yin H., Cheng W., Cheng J. (2021). Regulating
Fe-spin state by atomically dispersed Mn-N in Fe-NC catalysts with
high oxygen reduction activity. Nat. Commun..

[98] Huang Z. F., Wang J., Peng Y., Jung C. Y., Fisher A., Wang X. (2017). Design of efficient
bifunctional oxygen reduction/evolution electrocatalyst:
recent advances and perspectives. Adv. Energy
Mater..

[99] Ghora S., Chakraborty R., Bag S., Kumar M. M., Raj C. R. (2025). Transition
metal phosphide-based oxygen electrocatalysts for aqueous zinc–air
batteries. Chem. Commun..

[100] Kangasniemi K. H., Condit D., Jarvi T. (2004). Characterization of
Vulcan electrochemically oxidized under simulated PEM fuel cell conditions. J. Electrochem. Soc..

[101] Li L., Xing Y. (2006). Electrochemical durability
of carbon nanotubes in noncatalyzed
and catalyzed oxidations. J. Electrochem. Soc..

[102] Von Deak D., Biddinger E. J., Ozkan U. S. (2011). Carbon corrosion
characteristics of CN x nanostructures in acidic media and implications
for ORR performance. J. Appl. Electrochem..

[103] Fan J. G., Pan J. M., Wang H., Liu S., Zhan Y., Yan X. (2025). Research and progress in mitigating
carbon oxidation in air electrodes. Adv. Funct.
Mater..

[104] Worrell, E. ; Phylipsen, D. ; Einstein, D. ; Martin, N. Energy Use and Energy Intensity of the US Chemical Industry Energy Analysis Department Environmental Energy Technologies Division Ernest Orlando Lawrence Berkeley National Laboratory University of California: Berkeley, California; 2000.

[105] Parrino F., Roda G. C., Loddo V., Palmisano L. (2016). Elemental
Bromine Production by TiO2 Photocatalysis and/or Ozonation. Angew. Chem., Int. Ed..

[106] Grinbaum, B. ; Freiberg, M. Bromine. In Kirk-Othmer Encyclopedia of Chemical Technology; Wiley, 2000.

[107] Magazinovic R. S., Nicholson B. C., Mulcahy D. E., Davey D. E. (2004). Bromide
levels in natural waters: its relationship to levels of both chloride
and total dissolved solids and the implications for water treatment. Chemosphere.

[108] Yang Y., Komaki Y., Kimura S. Y., Hu H.-Y., Wagner E. D., Mariñas B. J., Plewa M. J. (2014). Toxic Impact of
Bromide and Iodide on Drinking Water Disinfected with Chlorine or
Chloramines. Environ. Sci. Technol..

[109] Jain D., Hightower J., Basu D., Gustin V., Zhang Q., Co A. C., Asthagiri A., Ozkan U. S. (2022). Highly active nitrogen–doped
carbon nanostructures
as electrocatalysts for bromine evolution reaction: A combined experimental
and DFT study. J. Catal..

[110] Hunt S. W., Roeselova M., Wang W., Wingen L., Knipping E., Tobias D., Dabdub D., Finlayson-Pitts B. (2004). Formation
of molecular bromine from the reaction of ozone with deliquesced NaBr
aerosol: Evidence for interface chemistry. J.
Phys. Chem. A.

[111] Yalçin H., Koç T., Pamuk V. (1997). Hydrogen and bromine
production from concentrated sea-water. Int.
J. Hydrogen Energy.

[112] Popat Y., Trudgeon D., Zhang C., Walsh F. C., Connor P., Li X. (2022). Carbon Materials as
Positive Electrodes
in Bromine-Based Flow Batteries. ChemPlusChem.

[113] Oh K., Weber A. Z., Ju H. (2017). Study of bromine
species crossover
in H2/Br2 redox flow batteries. Int. J. Hydrogen
Energy.

[114] Moussallem I., Jörissen J., Kunz U., Pinnow S., Turek T. (2008). Chlor-alkali electrolysis
with oxygen depolarized cathodes: history,
present status and future prospects. J. Appl.
Electrochem..

[115] Jain, D. Development of Alternative Materials to Replace Precious Metals in Sustainable Catalytic Technologies; The Ohio State University, 2019.

[116] Jain, D. ; Ozkan, U. S. Electrocatalytic applications of heteroatom-doped carbon nanostructures: thinking beyond PEM fuel cells. In Catalysis; RSC Publishing, 2020.

[117] Park J. S., Chen C., Wieder N. L., Vohs J. M., Gorte R. J. (2011). Electrolysis of HBr using molten, alkali-bromide electrolytes. Electrochim. Acta.

[118] Luttmer J. D., Konrad D., Trachtenberg I. (1985). Electrode
materials for hydrobromic acid electrolysis in Texas Instruments’
solar chemical converter. J. Electrochem. Soc..

[119] Petrov M.
M., Loktionov P. A., Konev D. V., Antipov A. E., Astafiev E. A., Vorotyntsev M. A. (2018). Evolution
of Anolyte Composition
in the Oxidative Electrolysis of Sodium Bromide in a Sulfuric Acid
Medium. Russ. J. Electrochem..

[120] Schuetz G. H., Fiebelmann P. J. (1980). Electrolysis of hydrobromic acid. Int. J. Hydrogen Energy.

[121] Kondo W., Mizuta S., Oosawa Y., Kumagai T., Fujii K. (1983). Decomposition of hydrogen bromide
or iodide by gas phase electrolysis. Bull. Chem.
Soc. Jpn..

[122] Janssen L. J. J., Hoogland J. G. (1970). Mechanism of bromine evolution at
a graphite electrode. Electrochim. Acta.

[123] Bommaraju, T. V. ; Lüke, B. ; O’Brien, T. F. ; Blackburn, M. C. Chlorine. In Kirk-Othmer Encyclopedia of Chemical Technology; Wiley, 2000.

[124] Tang L., Lu W., Zhang H., Li X. (2022). Progress and
Perspective of the Cathode Materials towards Bromine-Based Flow Batteries. Energy Mater. Adv..

[125] Vos J. G., Venugopal A., Smith W. A., Koper M. T. (2020). Competition
and selectivity during parallel evolution of bromine, chlorine and
oxygen on IrOx electrodes. J. Catal..

[126] Xu J., Georgescu N. S., Scherson D. A. (2014). The oxidation of bromide on platinum
electrodes in aqueous acidic solutions: electrochemical and in situ
spectroscopic studies. J. Electrochem. Soc..

[127] Rana M., Stoppiello C. T., He Q., Peng X., Alghamdi N., Huang Y., Gentle I. R., Luo B. (2023). Tin Modified
Carbon Nanofibers as an Effective Catalytic Electrode for Bromine
Redox Reactions in Static Zinc-bromine Batteries. Batteries Supercaps.

[128] Chen C., Liu T., Pu Z., Chen Z., Zhang X., Huang Q., Al-Enizi A. M., Nafady A., Zhang G., Sun S. (2024). Unveiling the Geometric
Site Dependence
of Co-Based Spinel Oxides in the Halogen Evolution Reaction. Adv. Sustainable Syst..

[129] Li L., Li R., Zhou S., Xu W., Li Y., Zhang J., Gao L., Pu X. (2024). Core-shell
Ni/NiO heterostructures
as catalytic cathodes enabling high-performance zinc bromine flow
batteries. Carbon Neutralization.

[130] Lai Q., Liu S., Jiang H., Zhang J., Zhou Z., Wang J., Wang Q., Wang Q. (2024). Urchin-Like Mesoporous
TiN Hollow Sphere Enabling Promoted Electrochemical Kinetics of Bromine-Based
Flow Batteries. Small.

[131] Jung H., Lee J., Park J., Shin K., Kim H. T., Cho E. (2023). A Mesoporous Tungsten Oxynitride
Nanofibers/Graphite Felt Composite Electrode with High Catalytic Activity
for the Cathode in Zn-Br Flow Battery. Small.

[132] Xiang H. X., Tan A. D., Piao J. H., Fu Z. Y., Liang Z. X. (2019). Efficient nitrogen-doped carbon for
zinc–bromine
flow battery. Small.

[133] Jin C.-x., Lei H.-y., Liu M.-y., Tan A.-d., Piao J.-h., Fu Z.-y., Liang Z.-x., Wang H.-h. (2020). Low-dimensional
nitrogen-doped carbon for Br2/Br– redox reaction in zinc-bromine
flow battery. Chem. Eng. J..

[134] Ozkan, U. ; Mamtani, K. ; Jain, D. Heteroatom-doped carbon catalyst for electrocatalytic halogen production. U.S. Patent US12,404,592B2, 2025.

[135] Weber A.
Z., Mench M. M., Meyers J. P., Ross P. N., Gostick J. T., Liu Q. (2011). Redox flow
batteries: a review. J. Appl. Electrochem..

[136] Kafle A., Gupta D., Mehta S., Garg K., Nagaiah T. C. (2024). Recent
advances in energy-efficient chlorine production
via HCl electrolysis. J. Mater. Chem. A.

[137] Kintrup J., Millaruelo M., Trieu V., Bulan A., Mojica E. S. (2017). Gas diffusion
electrodes for efficient manufacturing
of chlorine and other chemicals. Electrochem.
Soc. Interface.

[138] Jin C., Nagaiah T. C., Xia W., Bron M., Schuhmann W., Muhler M. (2011). Polythiophene-Assisted Vapor Phase Synthesis of Carbon
Nanotube-Supported Rhodium Sulfide as Oxygen Reduction Catalyst for
HCl Electrolysis. ChemSusChem.

[139] Singh V., Adhikary S. D., Tiwari A., Mandal D., Nagaiah T. C. (2017). Sustainable non-noble metal bifunctional
catalyst for
oxygen-depolarized cathode and Cl2 evolution in HCl electrolysis. Chem. Mater..

[140] Gupta D., Kafle A., Chaturvedi A., Nagaiah T. C. (2021). Recovery of High Purity Chlorine by Cu-Doped Fe2O3
in Nitrogen Containing Carbon Matrix: A Bifunctional Electrocatalyst
for HCl Electrolysis. ChemElectroChem.

[141] Biddinger E. J., Modestino M. A. (2020). Electro-organic syntheses for green
chemical manufacturing. Electrochem. Soc. Interface.

[142] Chen B., Dingerdissen U., Krauter J., Rotgerink H. L., Rotgerink H. L., Möbus K., Ostgard D., Panster P., Riermeier T., Seebald S., Tacke T. (2005). New developments in
hydrogenation catalysis particularly in synthesis of fine and intermediate
chemicals. Appl. Catal., A.

[143] Zeng Y., Zhao M., Zeng H., Jiang Q., Ming F., Xi K., Wang Z., Liang H. (2023). Recent progress
in advanced catalysts for electrocatalytic hydrogenation of organics
in aqueous conditions. Escience.

[144] Capdevila-Cortada M. (2019). Electrifying the haber–bosch. Nat. Catal..

[145] Sideri I. K., Tagmatarchis N. (2020). Noble-metal-free doped carbon nanomaterial
electrocatalysts. Chem. – Eur. J..

[146] Li J., Zan W.-Y., Kang H., Dong Z., Zhang X., Lin Y., Mu Y.-W., Zhang F., Zhang X.-M., Gu J. (2021). Graphitic-N
highly doped graphene-like carbon: A superior metal-free catalyst
for efficient reduction of CO2. Appl. Catal.,
B.

[147] Wu J., Liu M., Sharma P. P., Yadav R. M., Ma L., Yang Y., Zou X., Zhou X.-D., Vajtai R., Yakobson B. I. (2016). Incorporation
of nitrogen defects for efficient
reduction of CO2 via two-electron pathway on three-dimensional graphene
foam. Nano Lett..

[148] Wu J., Ma S., Sun J., Gold J. I., Tiwary C., Kim B., Zhu L., Chopra N., Odeh I. N., Vajtai R. (2016). A metal-free electrocatalyst
for carbon dioxide reduction to multi-carbon
hydrocarbons and oxygenates. Nat. Commun..

[149] Ren Y., Yu C., Tan X., Wei Q., Wang Z., Ni L., Wang L., Qiu J. (2022). Strategies
to activate inert nitrogen
molecules for efficient ammonia electrosynthesis: current status,
challenges, and perspectives. Energy Environ.
Sci..

[150] Martín A. J., Shinagawa T., Pérez-Ramírez J. (2019). Electrocatalytic
reduction of nitrogen: from Haber-Bosch to ammonia artificial leaf. Chem.

[151] Soloveichik G. (2019). Electrochemical synthesis of ammonia as a potential
alternative to the Haber–Bosch process. Nat. Catal..

[152] Yang X., Nash J., Anibal J., Dunwell M., Kattel S., Stavitski E., Attenkofer K., Chen J. G., Yan Y., Xu B. (2018). Mechanistic insights
into electrochemical nitrogen reduction reaction on vanadium nitride
nanoparticles. J. Am. Chem. Soc..

[153] Fernández C. A., Chapman O., Brown M. A., Alvarez-Pugliese C. E., Hatzell M. C. (2024). Achieving decentralized, electrified, and decarbonized
ammonia production. Environ. Sci. Technol..

[154] Qing G., Ghazfar R., Jackowski S. T., Habibzadeh F., Ashtiani M. M., Chen C.-P., Smith M. R., Hamann T. W. (2020). Recent advances and challenges of
electrocatalytic N2 reduction to ammonia. Chem.
Rev..

[155] Ferree M., Gunduz S., Kim J., LaRosa R., Khalifa Y., Co A. C., Ozkan U. S. (2023). Enhanced N2 activation
on a composite Co3Mo3N nitride and La0. 6Sr0. 4Co0. 2Fe0. 8O3 perovskite
cathode for high-temperature electrochemical ammonia synthesis. ACS Sustainable Chem. Eng..

[156] Gunduz S., Deka D. J., Ozkan U. S. (2020). A review
of the
current trends in high-temperature electrocatalytic ammonia production
using solid electrolytes. J. Catal..

[157] Gunduz S., Deka D. J., Ferree M., Kim J., Millet J.-M. M., Co A. C., Ozkan U. S. (2022). Composite cathodes
with oxide and nitride phases for high-temperature electrocatalytic
ammonia production from nitrogen and water. ECS Adv..

[158] Deng J., Iñiguez J. A., Liu C. (2018). Electrocatalytic nitrogen
reduction at low temperature. Joule.

[159] Chen S., Perathoner S., Ampelli C., Mebrahtu C., Su D., Centi G. (2017). Room-temperature
electrocatalytic synthesis of NH3
from H2O and N2 in a gas–liquid–solid three-phase reactor. ACS Sustainable Chem. Eng..

[160] Chen S., Perathoner S., Ampelli C., Mebrahtu C., Su D., Centi G. (2017). Electrocatalytic
synthesis of ammonia at room temperature
and atmospheric pressure from water and nitrogen on a carbon-nanotube-based
electrocatalyst. Angew. Chem., Int. Ed..

[161] Liu Y., Su Y., Quan X., Fan X., Chen S., Yu H., Zhao H., Zhang Y., Zhao J. (2018). Facile ammonia synthesis
from electrocatalytic N2 reduction under ambient conditions on N-doped
porous carbon. ACS Catal..

[162] Gupta D., Kafle A., Kaur S., S Thomas T., Mandal D., Nagaiah T. C. (2023). Selective electrochemical
conversion
of N2 to NH3 in neutral media using B, N-containing carbon with a
nanotubular morphology. ACS Appl. Mater. Interfaces.

[163] Zhao C., Zhang S., Han M., Zhang X., Liu Y., Li W., Chen C., Wang G., Zhang H., Zhao H. (2019). Ambient electrosynthesis
of ammonia on a biomass-derived nitrogen-doped
porous carbon electrocatalyst: contribution of pyridinic nitrogen. ACS Energy Lett..

[164] Yuan L.-P., Wu Z.-Y., Jiang W.-J., Tang T., Niu S., Hu J.-S. (2020). Phosphorus-doping
activates carbon nanotubes for efficient
electroreduction of nitrogen to ammonia. Nano
Res..

[165] Zhang L.-H., Yu F., Shiju N. R. (2021). Carbon-based catalysts
for selective electrochemical nitrogen-to-ammonia conversion. ACS Sustainable Chem. Eng..

[166] Kong J., Choi J., Park H. S. (2023). Advantages
and limitations
of different electrochemical NH3 production methods under ambient
conditions: a review. Curr. Opin. Electrochem..

[167] Kolen M., Ripepi D., Smith W. A., Burdyny T., Mulder F. M. (2022). Overcoming nitrogen reduction to
ammonia detection
challenges: the case for leapfrogging to gas diffusion electrode platforms. ACS Catal..

[168] Akhade S. A., Singh N., Gutiérrez O. Y., Lopez-Ruiz J., Wang H., Holladay J. D., Liu Y., Karkamkar A., Weber R. S., Padmaperuma A. B. (2020). Electrocatalytic hydrogenation of biomass-derived organics: a review. Chem. Rev..

[169] Liu C., Wu Y., Zhao B., Zhang B. (2023). Designed nanomaterials
for electrocatalytic organic hydrogenation using water as the hydrogen
source. Acc. Chem. Res..

[170] Kundu B. K., Sun Y. (2024). Electricity-driven
organic hydrogenation
using water as the hydrogen source. Chem. Sci..

[171] Koh K., Sanyal U., Lee M. S., Cheng G., Song M., Glezakou V. A., Liu Y., Li D., Rousseau R., Gutierrez O. Y. (2020). Electrochemically Tunable Proton-Coupled Electron
Transfer in Pd-Catalyzed Benzaldehyde Hydrogenation. Angew. Chem., Int. Ed..

[172] Serrà A., Artal R., Pozo M., Garcia-Amorós J., Gómez E. (2020). Simple Environmentally-Friendly Reduction
of 4-Nitrophenol. Catalysts.

[173] Zheng M., Zhang J., Wang P., Jin H., Zheng Y., Qiao S. Z. (2024). Recent advances in electrocatalytic
hydrogenation reactions on copper-based catalysts. Adv. Mater..

[174] Gong W., Ma J., Chen G., Dai Y., Long R., Zhao H., Xiong Y. (2025). Unlocking the catalytic
potential of heterogeneous nonprecious metals for selective hydrogenation
reactions. Chem. Soc. Rev..

[175] Pota F., de Oliveira M. A. C., Schroeder C., Rafferty A., De Castro C., Rault L., Behan J. A., Barrière F., Colavita P. E. (2025). Electrocatalytic hydrogenation of
unsaturated organics using Mo and W porous carbon-encapsulated nanostructures:
impact of metal type on properties and performances. J. Mater. Chem. A.

[176] Sun J., Ding Y., He D., Qiu X., Luo C., Jiang P. (2025). Carbon felt by acid treatment as a highly active metal-free electrocatalyst
for the selective hydrogenation of cinnamaldehyde to hydrocinnamaldehyde. Catal. Sci. Technol..

[177] Song Z., Yang R., Liu X., Zhang B., Wu Y. (2024). An Organic Molecular Mimetic Metal-Free
Heterogeneous Catalyst for
Electrocatalytic Alkyne Semihydrogenation. Angew.
Chem., Int. Ed..

[178] Grammenos A. O., Andre R. F., Saldaña F. I., Kamra M., Antonietti M., Odziomek M. (2025). Harnessing the Electrochemical
Hydrogen Storage Capability of N-Doped Carbons for Metal-Free Hydrogenations. ACS Catal..

[179] Ma R., Lin G., Zhou Y., Liu Q., Zhang T., Shan G., Yang M., Wang J. (2019). A review of
oxygen
reduction mechanisms for metal-free carbon-based electrocatalysts. npj Comput. Mater..

[180] Yuan Y., Li M., Bai Z., Jiang G., Liu B., Wu T., Chen Z., Amine K., Lu J. (2019). The absence
and importance of operando techniques for metal-free catalysts. Adv. Mater..

